# Ensemble genomic analysis in human lung tissue identifies novel genes for chronic obstructive pulmonary disease

**DOI:** 10.1186/s40246-018-0132-z

**Published:** 2018-01-15

**Authors:** Jarrett D. Morrow, Michael H. Cho, John Platig, Xiaobo Zhou, Dawn L. DeMeo, Weiliang Qiu, Bartholome Celli, Nathaniel Marchetti, Gerard J. Criner, Raphael Bueno, George R. Washko, Kimberly Glass, John Quackenbush, Edwin K. Silverman, Craig P. Hersh

**Affiliations:** 1 Channing Division of Network Medicine, Brigham and Women’s Hospital, 181 Longwood Avenue, Boston, MA 02115 USA; 2 Division of Pulmonary and Critical Care Medicine, Brigham and Women’s Hospital, Boston, MA 02115 USA; 3 Department of Biostatistics and Computational Biology, Dana-Farber Cancer Institute, Boston, MA 02115 USA; 4 Division of Pulmonary and Critical Care Medicine, Temple University, Philadelphia, PA 19140 USA; 5 Division of Thoracic Surgery, Brigham and Women’s Hospital, Boston, MA 02115 USA

**Keywords:** eQTL, Expression QTL, Integrative genomics, Network medicine, Ensemble methods, Bayesian methods

## Abstract

**Background:**

Genome-wide association studies (GWAS) have identified single nucleotide polymorphisms (SNPs) significantly associated with chronic obstructive pulmonary disease (COPD). However, many genetic variants show suggestive evidence for association but do not meet the strict threshold for genome-wide significance. Integrative analysis of multiple omics datasets has the potential to identify novel genes involved in disease pathogenesis by leveraging these variants in a functional, regulatory context.

**Results:**

We performed expression quantitative trait locus (eQTL) analysis using genome-wide SNP genotyping and gene expression profiling of lung tissue samples from 86 COPD cases and 31 controls, testing for SNPs associated with gene expression levels. These results were integrated with a prior COPD GWAS using an ensemble statistical and network methods approach to identify relevant genes and observe them in the context of overall genetic control of gene expression to highlight co-regulated genes and disease pathways. We identified 250,312 unique SNPs and 4997 genes in the cis(local)-eQTL analysis (5% false discovery rate). The top gene from the integrative analysis was *MAPT*, a gene recently identified in an independent GWAS of lung function. The genes *HNRNPAB* and *PCBP2* with RNA binding activity and the gene *ACVR1B* were identified in network communities with validated disease relevance.

**Conclusions:**

The integration of lung tissue gene expression with genome-wide SNP genotyping and subsequent intersection with prior GWAS and omics studies highlighted candidate genes within COPD loci and in communities harboring known COPD genes. This integration also identified novel disease genes in sub-threshold regions that would otherwise have been missed through GWAS.

**Electronic supplementary material:**

The online version of this article (10.1186/s40246-018-0132-z) contains supplementary material, which is available to authorized users.

## Background

Chronic obstructive pulmonary disease (COPD) is characterized by progressive airflow obstruction accompanied by chronic inflammation. It is a major cause of morbidity and mortality worldwide [1]. Although environmental exposures such as cigarette smoking are risk factors, a genetic component to susceptibility has been observed [2–5]. Multiple genome-wide association studies (GWAS) have identified loci associated with COPD susceptibility across various populations [6–9]. However, most of these associations have small effect sizes, so there are likely additional COPD genes to be discovered. Understanding the gene regulatory implications of the significant and sub-genome-wide significant (sub-threshold) GWAS variants in lung tissue may identify genes and loci relevant to COPD for future validation experiments.

Prioritization of previously identified genomic loci enhances the molecular understanding of complex disease [10, 11]. Additionally, sub-threshold genetic loci may play a role in complex diseases [12] such as COPD, as they likely carry a significant biological signal and may reach significance in later higher powered studies. Increasing the power to identify additional associations often requires a much larger sample size [13], which greatly increases study expense. Integration with omics data can provide insight into the regulatory effects of these variants [12, 14, 15], without increasing sample size. Expression quantitative trait locus (eQTL) analysis tests the association between genetic variants and gene expression and can point to relevant single nucleotide polymorphisms (SNPs) and genes within GWAS loci [15–17] using the observation that trait-associated SNPs are likely to be eQTLs/eSNPs [17] and/or have gene regulatory implications [18].

In this study of genetic control of gene expression, we performed eQTL analysis in lung tissue samples from severe COPD cases and ex-smoker controls and integrated the findings with results from a prior GWAS [8]. We used the Bayesian method Sherlock [19] to identify genes having collective associations within the significant and sub-threshold GWAS SNPs. To observe these genes in the overall context of genetic control of gene expression, we constructed a bipartite network and identified communities [20] harboring the Sherlock-derived genes. We observed that some of these communities contained differentially expressed genes and genes with CpG sites differentially methylated by COPD status. This integration of previous omics studies hones in on the communities demonstrating greater relevance to COPD.

The central hypothesis of this study is that sub-threshold GWAS SNPs, in addition to genome-wide significant SNPs, both influence gene expression and confer disease susceptibility through effects better observed using network and integrative statistical methods. The foundation of this study is the aggregation of the gene expression signals from SNPs identified in prior GWAS, both significant and sub-threshold, using regulatory evidence via an ensemble Bayesian and network approach. This integrative method extracts the additional genetic and genomic signals contained in the sub-threshold SNPs by combining evidence across genotyping, gene expression and DNA methylation datasets and highlights novel genes and loci within regions that may not have been identified through GWAS. This motivates hypotheses regarding the biological role of these findings in disease and informs selection of targets for further functional investigations.

## Results

**Fig. 1:**
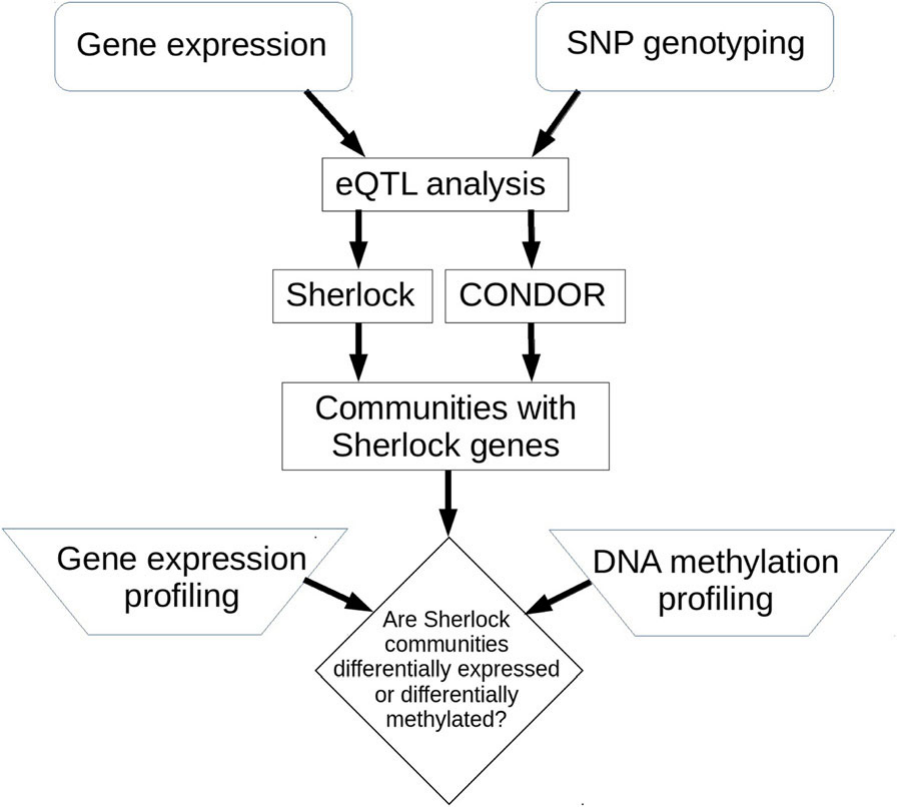
Graphical overview of the study methods and process. The cis- and trans-eQTLs identified in lung tissue were integrated with prior GWAS using Bayesian and network methods. The network communities identified were interrogated for evidence of differential gene expression and differential DNA methylation by COPD status

Gene expression data were available for lung tissue samples from 86 severe COPD cases (mean FEV_1_ 26.4% predicted) and 31 controls with normal spirometry, all Caucasians (Additional file 1: Table S1). There were no significant differences between cases and controls by sex or age. The cases had higher lifetime smoking intensity in pack-years and quit smoking on average 8.7 fewer years in the past (*p* = 0.0006). We identified eQTLs using the gene expression and imputed genotyping data and integrated them with prior GWAS and omics studies using an ensemble approach of statistical and network methods (Fig. 1).

Using the lung tissue gene expression profiling and imputed genotyping data from the cases and controls, we performed cis- and trans-eQTL analysis (see the “Methods” section). We identified 347,251 significant cis-eQTL results (FDR < 5%) out of 55,550,191 total tests. Within these results, there were 250,312 unique cis-eQTL SNPs (eSNPs) and 5878 unique eQTL genes (eGenes, 4997 gene symbols) (Additional file 1: Table S2). This represents 4.2% of the SNPs and 24% of the expression probes tested. The trans results contain 8519 significant results (FDR < 5%), out of 146,665,850,054 total tests, with 6930 unique eSNPs and 451 unique eGenes (434 gene symbols) (Additional file 1: Table S3).

**Fig. 2:**
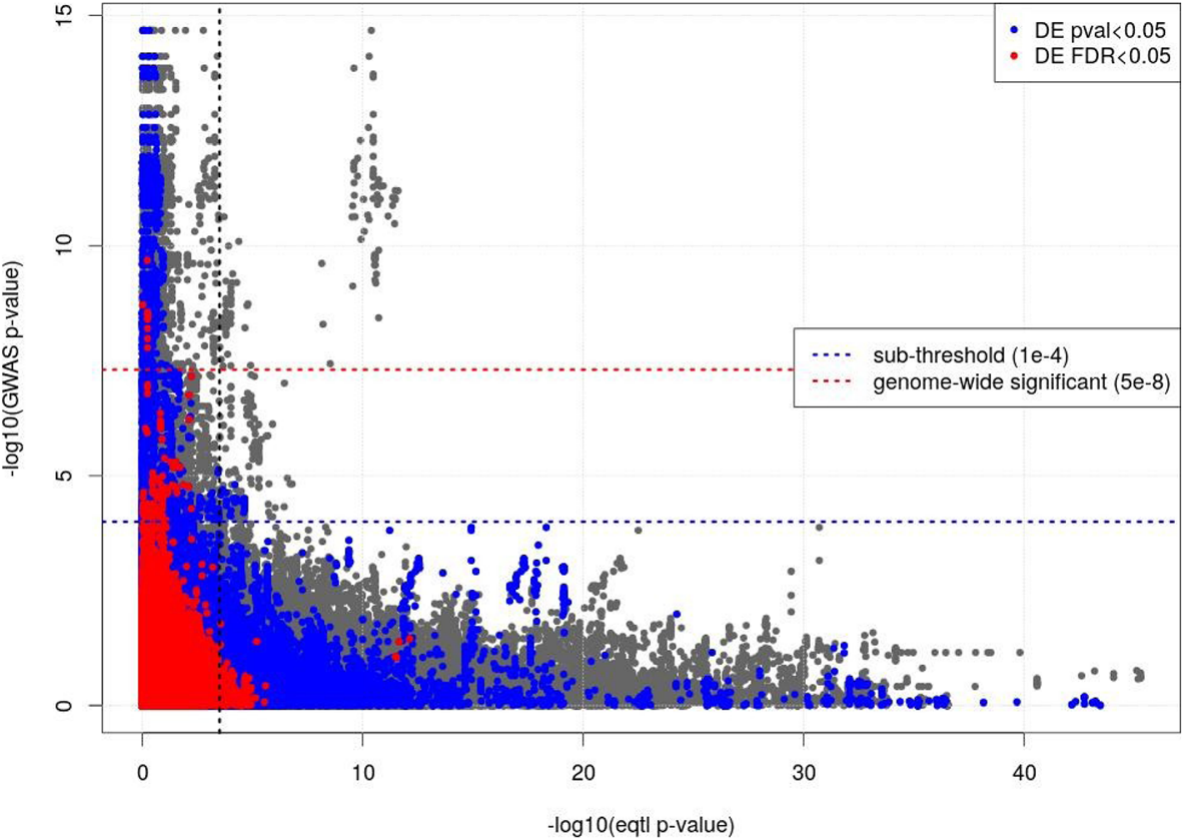
Plot of COPD GWAS *p* values vs. the cis-eQTL *p* values. Each point in the plot represents a cis-eQTL result with an rsID found in the prior GWAS. GWAS *p* values (*y* axis) are plotted against the expression QTL *p* values (*x* axis). A vertical dotted line indicates the threshold of significance (FDR < 5%) for the eQTL. Horizontal lines delineate genome-wide significant (red) and sub-threshold (blue) GWAS *p* values. The significant (red; FDR < 5%) and nominally significant (blue; *p* < 0.05) eGenes from gene expression profiling in COPD lung tissue are highlighted

We intersected the significant cis-eQTL results with the GWAS at a suggestive level of significance (*p* < 10^−4^) [8] and observed that 292 of these 1847 significant and sub-threshold GWAS SNPs were eSNPs (4.3 fold enrichment, hypergeometric *p* value < 0.00001). The top intersection results are shown in (Additional file 1: Table S4). Regional genomic plots of significant cis-eQTLs (FDR < 5%) for 5 of these 13 loci highlight the regulatory information for the top eSNPs and SNPs in linkage disequilibrium (LD) (Additional file 1: Figures S1–S5). Two of the eSNPs from (Additional file 1: Table S4) are located within the associated eGene (rs1504550-*IREB2* and rs2252518-*ACVR1B*; Additional file 1: Figures S1 and S2). Two others (rs12461383-*C19orf54* and rs11852372-*CHRNA5*; Additional file 1: Figures S3 and S4) are in promoter flanking and transcription factor binding regions within DNase hypersensitivity (DHS) sites. The last eSNP (rs151321-*SULT1A2*; Additional file 1: Figure S5) is in LD (shaded in red) with several SNPs located in regulatory regions. To observe overall genetic control of gene expression in a disease context, we intersected all cis-eQTL results with the nominally significant GWAS SNPs (*p* < 0.05) [8] and plotted the *p* values from the two sets (Fig. 2). Each point in the plot represents an eQTL result (eSNP-eGene pair); prior COPD gene expression profiling results [21] are overlaid in color. We observed that eQTLs with COPD GWAS associations are generally not enriched for differentially expressed genes; regions with sub-threshold GWAS *p* values (*p* < 10^−4^) and significant eQTL *p* values lack differentially expressed genes (FDR < 5%). Therefore, we used additional statistical and network methods to extract the signal in these results, given this complex relationship between the disease and the genetic control of gene expression.

We integrated the nominally significant cis-eQTLs (*p* < 10^−3^) and trans-eQTLs (*p* < 10^−6^) with prior GWAS using the Bayesian method Sherlock [19], seeking genes with collective associations across the significant and sub-threshold GWAS results. The 438,536 SNPs common to the eQTL, GWAS, and GWAS permutation data were the basis for this integrative analysis. A total of 50 Sherlock results had *p* values < 10^−3^ (Table 1, Additional file 2: Table S5). This *p* value threshold corresponds to a LBF (logarithm of Bayes factor) sum of 1.94. Of the 50 genes identified, 13 were previously found in the intersection between cis-eQTLs and GWAS (*p* < 10^−4^) results. Several genes have been identified in previous COPD GWAS studies. We repeated the Sherlock analysis using the eQTL results from GTEx V7 (using the same *p* value thresholds) and observed the results for these top 50 genes (Table 1). We further sought to place our 50 Sherlock-derived genes in the context of overall genetic control of gene expression using network methods, since co-regulated genes may have shared function. This process has the potential to reveal additional COPD genes of interest.

**Table 1:** COPD genes identified in the Sherlock analysis

Gene symbol	Total LBF score	Sherlock *p* value	Differentially expressed probe (*p* < 0.05)	Differentially methylated site (*p* < 0.05, effect > 5%)	GTEx V7 (cis-only, LBF score)	GTEx V7 (cis-only, *p* value)
*MAPT*	7.65	6.91E-07	Yes	No	7.31	6.41E-07
*LRRC37A4*	7.46	6.91E-07	No	No	2.13	1.8E-3
*C17orf69*	7.38	6.91E-07	Yes	No	2.39	1.3E-3
*IREB2* ^*^	6.40	6.91E-07	No	No	4.37	5.89E-05
*C19orf54* ^*^	5.45	5.53E-06	No	No	5.01	1.79E-05
*ACVR1B* ^*^	5.40	5.53E-06	No	Yes	4.77	2.82E-05
*EIF3CL* ^*^	4.45	1.94E-05	No	No	N/A	N/A
*TUFM* ^*^	4.29	2.49E-05	Yes	No	5.31	8.97E-06
*FAM13A*	4.09	3.60E-05	No	No	5.69	3.84E-06
*PCBP2*	3.97	4.43E-05	No	No	N/A	N/A
*CYP2B7* ^*^	3.87	5.67E-05	No	No	N/A	N/A
*SULT1A1* ^*^	3.81	6.08E-05	Yes	No	5.38	8.97E-06
*SULT1A2* ^*^	3.80	6.08E-05	Yes	Yes	5.01	1.79E-05
*TIGD2*	3.58	7.88E-05	No	No	2.48	1.10E-03
*CHRNA5* ^*^	3.37	1.05E-04	No	No	5.27	1.15E-05
*BZRAP1*	3.27	1.20E-04	No	Yes	−0.20	7.28E-01
*GPX8* ^*^	3.25	1.20E-04	No	No	N/A	N/A
*TEKT3*	3.19	1.31E-04	No	No	0.00	1.59E-01
*SNRPB*	3.06	1.63E-04	No	No	0.00	1.61E-01
*ZNF652*	3.03	1.69E-04	No	No	−0.02	2.69E-01
*AHSA2* ^*^	2.86	2.14E-04	No	No	2.22	1.63E-03
*CDH23*	2.82	2.21E-04	No	Yes	0.01	1.38E-01
*NOP2*	2.69	2.99E-04	No	No	N/A	N/A
*AASDH*	2.68	3.06E-04	No	No	2.45	1.14E-03
*DAGLA*	2.68	3.06E-04	No	No	N/A	N/A
*IFI27L2* ^*^	2.65	3.24E-04	No	No	2.90	5.98E-04
*APIP*	2.60	3.48E-04	No	No	2.52	1.03E-03
*AXIN2*	2.59	3.55E-04	No	No	−0.04	4.10E-01
*WDR47*	2.49	4.20E-04	Yes	No	N/A	N/A
*C4orf33*	2.41	4.74E-04	No	No	1.68	3.71E-03
*HNRNPAB*	2.34	5.23E-04	No	No	N/A	N/A
*GFPT1*	2.33	5.24E-04	Yes	No	−0.02	2.04E-01
*LOC644172*	2.32	5.30E-04	No	No	N/A	N/A
*SNORD25*	2.25	5.86E-04	No	No	N/A	N/A
*PPAT*	2.23	6.06E-04	Yes	No	−0.03	3.22E-01
*FBRSL1*	2.23	6.08E-04	No	No	−0.09	5.90E-01
*FSTL5*	2.22	6.14E-04	No	No	N/A	N/A
*SMG6*	2.18	6.44E-04	No	Yes	−0.01	1.85E-01
*CHIAP2*	2.09	7.58E-04	No	No	N/A	N/A
*RPL23A*	2.07	7.77E-04	Yes	No	N/A	N/A
*C2orf74* ^*^	2.06	7.85E-04	No	No	N/A	N/A
*CTSH*	2.04	8.17E-04	No	No	1.40	5.59E-03
*UBE2J1*	2.03	8.27E-04	Yes	No	N/A	N/A
*AEN*	2.01	8.50E-04	No	No	0.36	3.61E-02
*CUL1*	2.00	8.88E-04	No	No	−0.02	3.02E-01
*DSP*	2.00	8.91E-04	No	No	1.51	4.77E-03
*MYCN*	1.97	9.32E-04	No	No	−0.04	4.13E-01
*TRIM4*	1.96	9.43E-04	Yes	No	1.57	4.37E-03
*ZNF57*	1.94	9.64E-04	No	No	−0.02	2.16E-01
*NARS2*	1.94	9.74E-04	No	No	2.89	6.10E-04

*LBF* logarithm of Bayes factor

*Gene identified in the cis-eQTL-GWAS intersection in (Additional file 1: Table S4)

We constructed a bipartite network using the cis- and trans-eQTLs with *p* value thresholds identical to those for Sherlock (cis: *p* < 10^−3^ and trans: *p* < 10^−6^). After all filtering steps (see the “Methods” section), 171,490 eSNPs and 11,348 eGenes were used in the construction of the network. The power-law nature of the degree distribution for this network is heavy-tailed (Additional file 1: Figure. S6) and similar to that seen in other bipartite eQTL networks [20], suggesting a scale-free structure characterized by the presence of hubs. We identified 250 communities within this network and focused on the 14 that contain Sherlock-derived genes (Table 2, Additional file 1: Table S6). We also examined two communities that contained putative interactors (*HMGB1* and *CD79A*) of genes near GWAS loci from our previous study [21]. These differentially expressed interactors were identified using gene expression profiling in lung tissue and in vitro, in vivo, and in silico datasets that identified genes with evidence of interaction with one of the three genes (*HHIP*, *FAM13A*, and *IREB2*) implicated by in-depth functional studies at COPD GWAS loci.

**Table 2:** CONDOR communities that contain Sherlock-derived genes or putative COPD GWAS gene interactors

Community ID	Sherlock or interactor gene(s)	Total SNPs	Sub-threshold SNPs	Total genes	Number of differentially expressed genes	Number of differentially methylated genes	Expression meta-*p* value	Methylation meta-*p* value
98^**^	*HMGB1*	143	0	4	3	1	2.26E-19	0.0028
113^**^	*CDH23*	489	0	12	2	1	0.0184	4.95E-05
135^**^	*CD79A*	1959	0	162	29	13	9.27E-11	4.27E-33
202^**^	*CHRNA5, HNRNPAB, IREB2, PCBP2*	293	57	17	4	1	0.0032	0.0026
218^**^	*ZNF652*	410	0	47	8	3	0.0017	4.85E-06
222^**^	*ACVR1B*	790	0	67	12	9	0.0003	8.23E-16
223^**^	*RPL23A*	509	4	32	10	6	3.26E-06	8.82E-18
20^*^	*WDR47*	476	0	6	3	0	0.0021	–
78^*^	*CHIAP2*	631	0	18	3	1	0.0907	0.0019
131	*AHSA2 C2orf74*	599	8	4	1	0	0.0634	–
161^*^	*SMG6*	633	0	18	1	2	0.3723	3.38E-09
179^*^	*DSP*	68	0	7	3	0	0.0060	–
181^*^	*FSTL5*	475	0	23	4	1	0.0503	0.0069
187^*^	*SNRPB*	178	0	14	1	2	0.5669	0.0001
210	*CTSH*	439	5	12	0	0	0.5741	–
249	*TRIM4*	555	0	11	3	0	0.0957	–

*Communities with either significant differential expression or differential methylation (*p* < 0.05)

**Communities with both significant differential expression and differential methylation (*p* < 0.05)

**Fig. 3:**
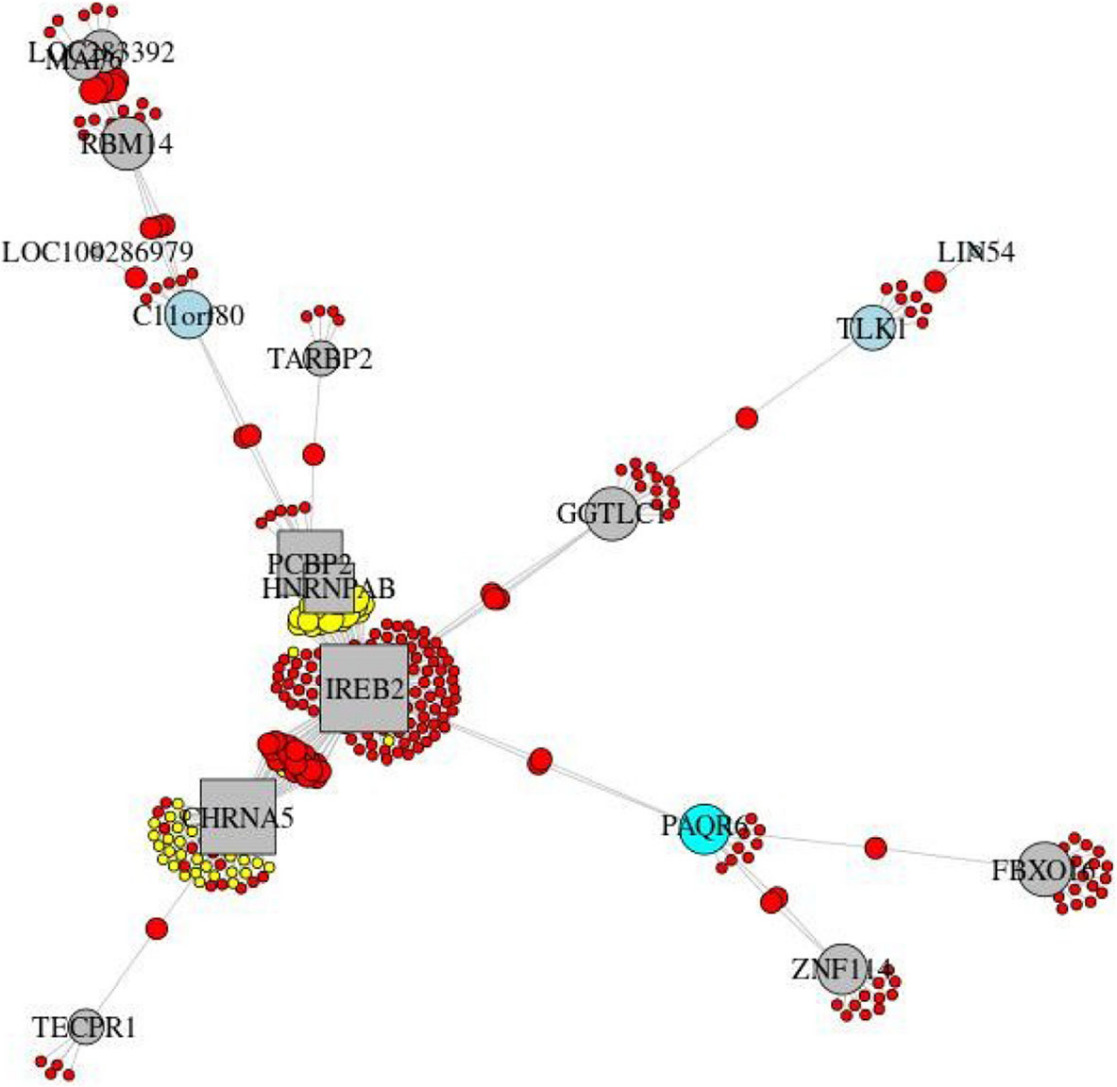
Community 202 from CONDOR analysis that contains the Sherlock-derived genes *CHRNA5*, *HNRNPAB*, *IREB2*, and *PCBP2*. Community genes are listed in (Additional file 1**:** Table S6**)**. (Red = SNP, yellow = SNP with GWAS *p* < 10^−4^, square = Sherlock gene, gray = gene, green = gene with differentially methylated site (*p* < 0.05 and effect > 5%), light blue = gene with differentially expressed probe (*p* < 0.05), and cyan = gene with differentially methylated site and differentially expressed probe)

**Fig. 4:**
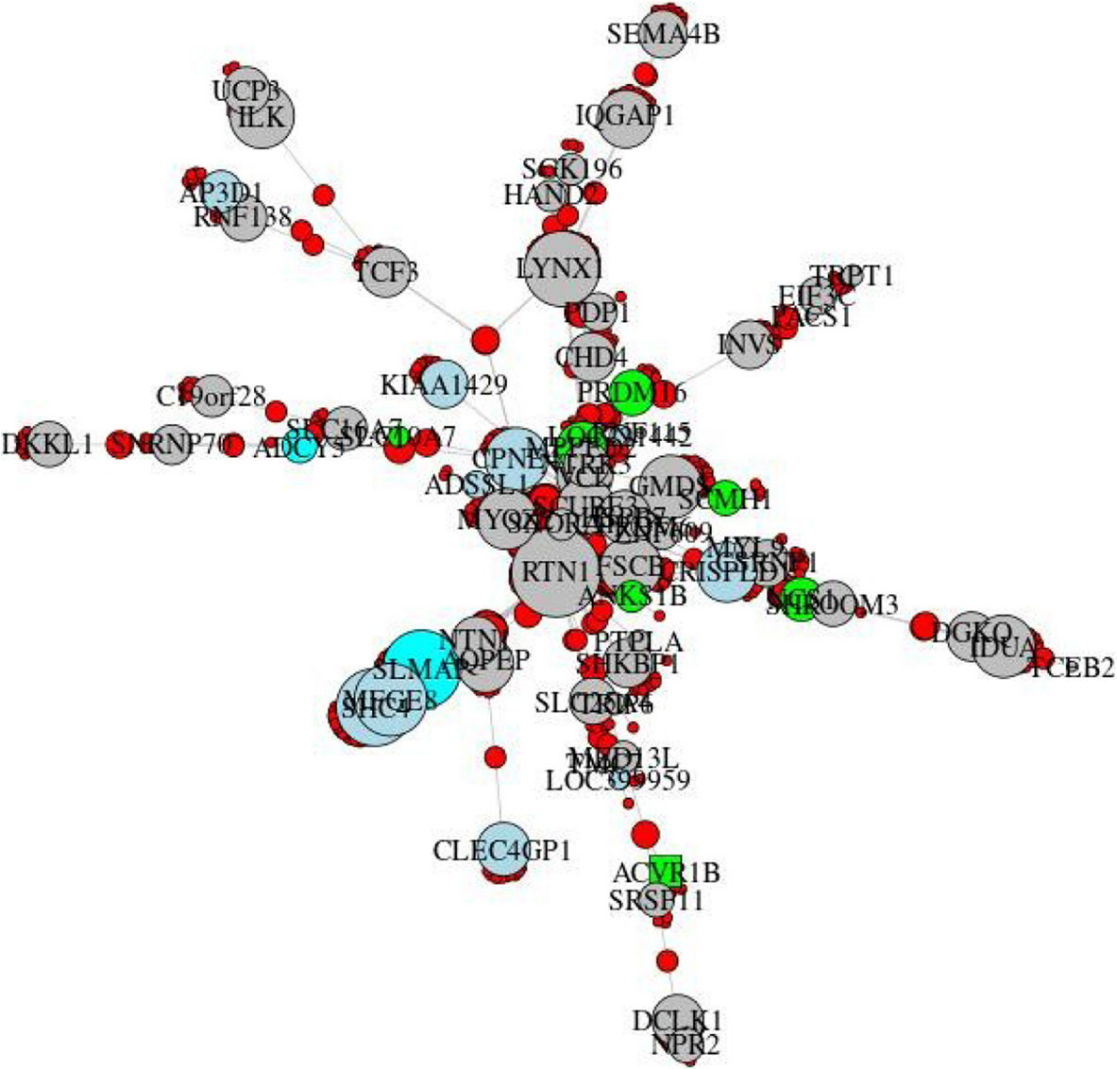
Community 222 from CONDOR analysis that contains the Sherlock-derived gene *ACVR1B*. Community genes are listed in (Additional file 1: Table S6**)**. (Red = SNP, yellow = SNP with GWAS *p* < 10^−4^**,** square = Sherlock gene, gray = gene, green = gene with differentially methylated site (*p* < 0.05 and effect > 5%), light blue = gene with differentially expressed probe (*p* < 0.05), and cyan = gene with differentially methylated site and differentially expressed probe)

**Fig. 5:**
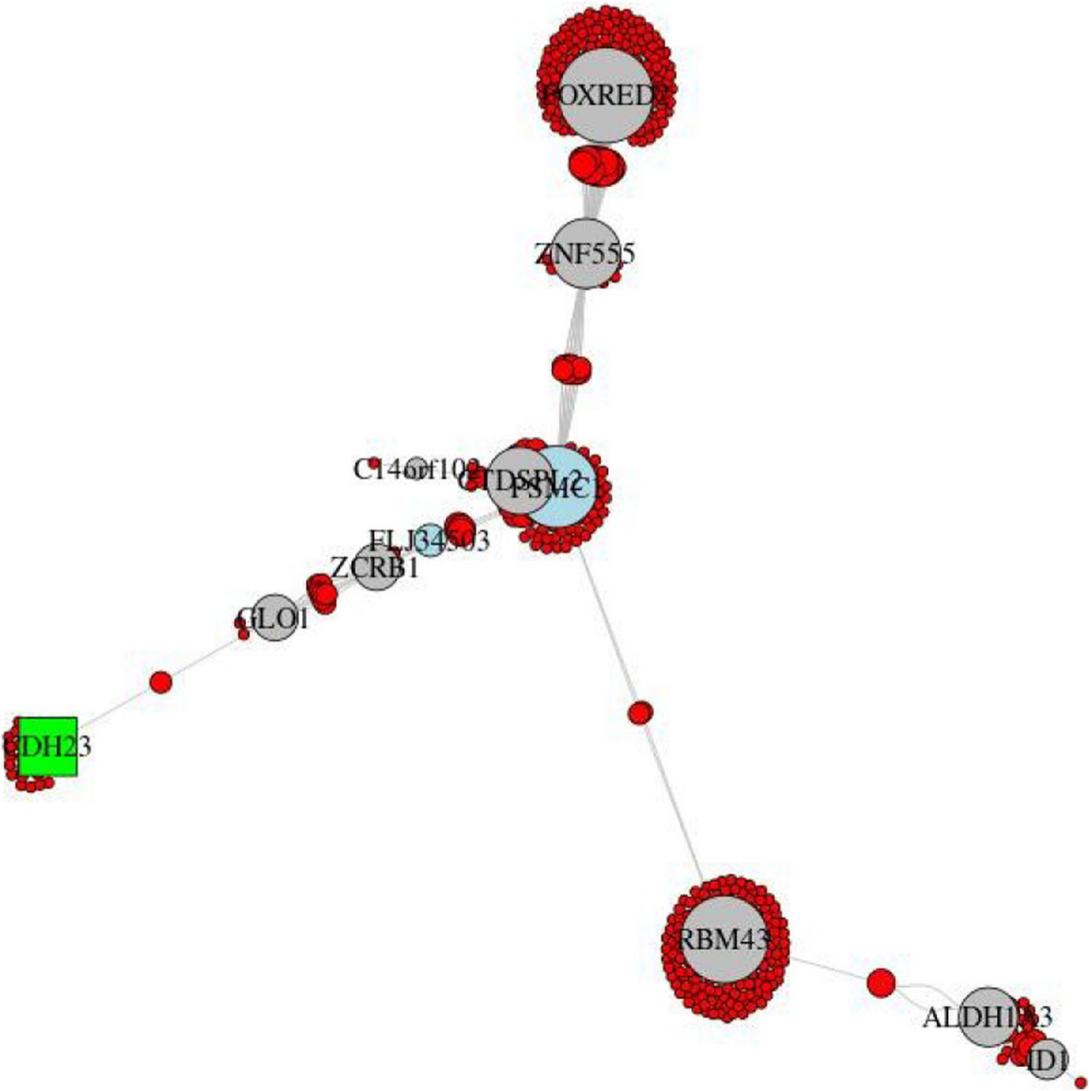
Community 113 from CONDOR analysis that contains the Sherlock-derived gene *CDH23*. The central genes *PSMC1* and *CTDSPL2* partially overlap and are obstructed in the figure. Community genes are listed in **(**Additional file 1: Table S6). (Red = SNP, yellow = SNP with GWAS *p* < 10^**−4**^, square = Sherlock gene, gray = gene, green = gene with differentially methylated site (*p* < 0.05 and effect > 5%), light blue = gene with differentially expressed probe (*p* < 0.05), cyan = gene with differentially methylated site and differentially expressed probe)

To validate the disease relevance of the communities, we calculated the differential expression and differential DNA methylation meta-analysis *p* values (see the “Methods” section) for these 16 communities. Seven communities were validated based on nominally significant (meta-*p* < 0.05) differential expression and differential methylation results (Table 2). These communities contain the Sherlock-derived genes C*DH23*, *CHRNA5*, *HNRNPAB*, *IREB2*, *PCBP2*, *ZNF652*, *ACVR1B*, and *RPL23A* (Figs. 3, 4, and 5 and Additional file 1: Figures S7–S8) or the interactors *HMGB1* and *CD79A* (Additional file 1: Figures S9–S10). There was significant pathway enrichment (FDR *q* value < 0.05) using ConsensusPathDB [22] for two validated communities (ID = 222:*ACVR1B* and ID = 135:*CD79A*) in Table 2 (Additional file 1: Table S7), highlighting cGMP-PKG signaling, focal adhesion, and actin and immune system-related pathways. Six of the nine remaining communities, which were lacking joint evidence, had either nominally significant differential expression or differential methylation.

## Discussion

Although many genome-wide significant loci from COPD GWAS were not eSNPs in lung tissue, we found that the sub-threshold GWAS findings are enriched in eSNPs. We also observed that eQTLs with GWAS associations did not have eGenes significantly differentially expressed in severe COPD cases vs controls, demonstrating the complex nature of genetic control of gene expression. We employed an ensemble approach involving Bayesian and network methods to investigate these eQTL results, which yielded 16 relevant bipartite communities. Based on the differential gene expression and/or differential DNA methylation of all of the genes or CpG sites within each community, we validated the disease relevance for 13 of these communities, highlighting potential COPD genes within the significant and sub-threshold GWAS results.

One of the seven communities (community 202) which was validated by both differential expression and DNA methylation contains two previously identified COPD GWAS genes located in a genome-wide significant region: *IREB2* (iron responsive element binding protein 2) and *CHRNA5* (cholinergic receptor nicotinic alpha 5 subunit) [23–25]. The product of *IREB2* is known to interact with mRNA to influence translation or degradation. Two other Sherlock-derived genes in community 202 also have putative RNA binding activity, *PCBP2* (poly(rC) binding protein 2) and *HNRNPAB* (heterogeneous nuclear ribonucleoprotein A/B). *PCBP2* plays a role in mRNA stability, and it has been suggested that deregulation of this stability may contribute to COPD pathogenesis [26]. A recent study of breast cancer highlighted the regulatory role of RNA binding by *PCBP3* (paralog of *PCBP1* along with *PCBP2*) on mRNA stability and induction of epithelial-mesenchymal transition (EMT) [27]. Additionally, *HNRNPAB* has been shown to induce EMT [28], a potential contributor to airway disease [29, 30]. Together, this suggests a role for this community in COPD pathogenesis. Community 222 contains the Sherlock-derived gene *ACVR1B* (activin A receptor type 1B), a gene identified in a previous eQTL study in blood and sputum in COPD [31]. *ACVR1B* was a sub-threshold finding in a GWAS of lung function in COPD [32] and was identified in our intersection of eQTLs with the sub-threshold GWAS of case-control status. The genes in community 222 were enriched for cGMP-PKG signaling, bacterial invasion of epithelial cells, and focal adhesion pathways [33], with possible relevance to COPD pathogenesis and exacerbations. Community 113 includes the Sherlock-derived gene *CDH23* (cadherin-related 23), involved in cell-cell adhesion and perhaps EMT as a calcium-dependent cell adhesion molecule [34]. This gene was contained within sub-threshold loci in GWAS of lung function decline [35], occupational asthma [36], and age at smoking initiation [37]. *DSP* (desmoplakin) was in a community (ID = 179) validated by differential expression but not differential methylation. *DSP* has been identified in a recent COPD GWAS meta-analysis [9] and in a study of interstitial lung disease [38]. Identifying this gene, which has only been highlighted in recent higher powered studies, supports our hypothesis that sub-threshold SNPs have the potential to confer disease susceptibility; genes in communities 222 and 113 may also be found significant in future GWAS.

The Sherlock analysis itself, prior to network integration, identified genes of interest that were not found through the simple intersection of eQTL and GWAS results. One of these genes, *MAPT* (microtubule associated protein tau), was previously found in a locus associated with extremes of lung function [39] and was suggestive in a recent COPD GWAS meta-analysis (*p* = 4.5 × 10^−3^) [9]. Genome-wide significant loci near *MAPT* were found to be associated with pulmonary fibrosis [38, 40]. In our previous gene expression profiling study, we observed a *MAPT* expression probe nominally differentially expressed (*p* < 0.05) in lung tissue of COPD cases vs. controls [21]. In the Sherlock analysis of the GTEx V7 results, we observed robust replication, with high scores from GTEx (LBF > 2.1) for eight of our top ten findings. Overall, 17 of the 35 genes that overlap our top 50 Sherlock genes attained a LBF of 1.94 or higher in the GTEx data. Trans-eQTL results are not available in GTEx (see the “Methods” section), preventing a complete replication of our findings, as the trans-eQTLs contributed important information to the COPD lung tissue Sherlock analysis. In addition, seven of the COPD lung tissue Sherlock genes were not included in the GTEx Sherlock input and eight other genes were not available in GTEx V7 eQTL data.

Four genes in a complex region on chromosome 16 associated with COPD in an exome array study [41] were identified in the Sherlock analysis and in the eQTL-GWAS intersection: *TUFM* (Tu translation elongation factor, mitochondrial), *EIF3CL* (eukaryotic translation initiation factor 3 subunit C like), *SULT1A1* (sulfotransferase family 1A member 1), and *SULT1A2* (sulfotransferase family 1A member 2). Nominal associations (*p* < 0.05) for *SULT1A2* were found in both previous gene expression profiling [21] and DNA methylation profiling [42] studies; nominal results for only gene expression were observed for *SULT1A1* and *TUFM*. Two genes in the Sherlock results, *CYP2B7* (cytochrome P450 family 2 subfamily B member 7, pseudogene) and *C19orf54* (chromosome 19 open reading frame 54), are located in another complex COPD locus on chromosome 19 [7]. Further efforts will be required to determine which of these genes is relevant for COPD pathogenesis.

In a previous gene expression profiling study [21], we identified several putative interactors of three known COPD GWAS genes (*HHIP*, *FAM13A*, and *IREB2)*. Communities harboring two of these interactors were identified in the current study. Both community 98 with *HMGB1* (high-mobility group box 1) and community 135 with *CD79A* (CD79a molecule) had evidence of differential expression and differential methylation. Additionally, there may also be a role for *HMGB1* in the development of EMT in airway epithelial cells [43].

Our study has several limitations. The omics datasets in this study were generated using homogenized lung tissue, so we could not determine the cellular specificity of the eQTLs, differential expression, and differential methylation. Studies in single lung cell types will address this cellular heterogeneity and provide validation of the findings. Our study focused on severe COPD and was enriched for subjects with emphysema and therefore may miss genes relevant for milder disease or other COPD phenotypes such as airway disease. Lastly, future integrative studies using these datasets will explore in more detail the gene regulatory impact of DNA methylation in lung tissue.

This study of the genetic control of gene expression in human lung has revealed potential genes of interest co-regulated with known COPD genes. The ensemble approach using statistical and network methods also pointed to specific genes in complex genomic regions found through prior GWAS, and genes within loci that would not meet strict thresholds for genome-wide significance, thereby extracting additional information from these results and supporting our hypothesis regarding the relevance of sub-threshold SNPs. We integrated three omics datasets, providing regulatory characterization of significant and sub-threshold GWAS variants, and highlighted genes for further functional investigation that may be involved in COPD pathogenesis. These genes would otherwise not have been identified through GWAS and could potentially meet the strict threshold for statistical significance in larger GWAS in COPD.

## Methods

### Study subjects

We collected lung tissue samples from former smokers undergoing thoracic surgery for lung transplantation, lung volume reduction surgery, or lung nodule resection at three medical centers; all subjects quit smoking at least 1 month prior to surgery [21, 42]. Distant normal tissue was sourced from lung nodule resection samples. The COPD subjects had severe airflow obstruction, with GOLD grade 3–4 spirometry (FEV1% predicted < 50% and FEV1/FVC < 0.7) and the controls had normal spirometry (FEV1% predicted ≥ 80% and FEV1/FVC ≥ 0.7). IRB approval was obtained at the three centers (Brigham and Women’s Hospital, Boston, MA; St. Elizabeth’s Hospital; Boston, MA; and Temple University Hospital, Philadelphia, PA), and subjects provided written informed consent.

### eQTL analysis

Microarray expression profiling was available for 111 cases and 40 controls [21] (GEO Series GSE76925). Of the 32,831 expression probes, 24,495 had genomic location information and were retained for integration with genotyping data. Genome-wide SNP genotyping data was obtained from lung tissue DNA using the HumanOmni2.5Exome-8 V1.0 BeadChip (Illumina, Inc., San Diego, CA) as previously described [21]. After quality control, genotypes were phased using SHAPEIT2 [44] and imputed using IMPUTE2 [45, 46] with the 1000 Genomes Phase3 V5 reference. The analyses were performed using only data from the Caucasian subjects. Data for markers with an imputation info metric > 0.5 and minor allele frequency > 5% were retained for the 117 Caucasian subjects that had both high-quality genotyping and gene expression data (86 cases and 31 controls; Additional file 1: Table S8). To account for population stratification, two principal components (PC) based on the Tracy-Widom statistic for the Caucasian population were retained [47]. Both cis- and trans-eQTL analyses were performed using the R/Bioconductor package Matrix eQTL (version 2.1.1) [48]. A total window size of 1 million bases was used for the cis analysis (500 kb upstream and downstream from the gene); trans analysis was performed genome-wide. This analysis identifies associations between genotype dosage and gene expression levels, adjusting for age, sex, pack-years of smoking, and the two ancestry PCs. An iterative method was used to determine the number of PCs for the matrix of expression values to add as covariates to mitigate batch effects [21]; 13 PCs were included in the eQTL analyses. An eQTL association result consists of an eGene (microarray expression probe) and eSNP pair.

### Integration using Sherlock

The Sherlock method performs genetic signature matching using a Bayesian statistical framework [19]. The hypothesis is that SNPs associated with expression of disease-relevant genes are also likely to influence disease risk and be identified through GWAS. Using Sherlock, we integrated the cis- and trans-eQTLs with all results from a published COPD GWAS [8]. Sherlock provides a total score for each gene, along with the score for each of the individual eQTL contributions. This total score is the sum of the LBFs (logarithm of Bayes factor) for each of these contributions. For interpretation of individual results, a value of 4.0 is typically required for significance. To output a *p* value, we created permuted GWAS results with similar linkage disequilibrium structure to the GWAS using the set of 379 EUR genotypes available in 1000 Genomes Phase1 V3 [49]. Specifically, we randomly permuted the case-control phenotypes 50 times as recommended in the Sherlock method (190 cases and 189 controls) and applied Plink2 [50] to calculate association *p* values for each iteration and used these results as inputs for Sherlock. Only overlapping SNPs (loci with rsIDs) present across the eQTL, GWAS, and permutation results were included in the analysis; minor allele frequencies for these markers were obtained from 1000 Genomes data. In the ensemble analysis, we applied a *p* value threshold of 10^−3^ to select a more significant set of Sherlock-derived genes for downstream analysis. We performed a replication of the Sherlock analysis using the GTEx V7 lung tissue eQTL results [51]. Only the GTEx markers found across the COPD GWAS [8] and our permutation results were included in the analysis. The GTEx project produced cis-eQTL results using a window of 1 million bases upstream and downstream. To align this Sherlock input with our study, we labeled eSNPs located 500 kb to 1 million bases from the gene transcription start site as trans-eQTLs.

### Network construction

A bipartite network was constructed using the cis- and trans-eQTLs. Network nodes are eGenes represented by their gene symbol annotation and eSNPs represented by their rsIDs. Edges only connect eSNPs to eGenes; no edges are present between pairs of eSNPs or pairs of eGenes. Only eSNPs represented in the GWAS were included in the network. Cis- or trans-eQTLs with only a single edge between an eSNP and eGene were excluded, since they did not create additional connections in the network. We identified communities within this bipartite network using the R package CONDOR [20] and visualized them using the R package igraph [52], with the Fruchterman-Reingold algorithm. A differential expression meta-analysis *p* value was computed for each community of interest. Specifically, the differential expression *p* values from prior expression profiling [21] for each expression probe annotated to genes in the community were combined using Fisher’s method via the R package metap. For differential DNA methylation, we used a similar approach based on prior methylation profiling results [42] for CpG sites annotated to genes in the community. In order to focus on CpG sites more likely to be biologically relevant, we required that the mean difference in methylation between cases and controls be greater than 5%.

### Regulatory annotation

The R package Sushi [53] was used with gene annotation and regulatory information from Ensemble BioMart [54] (CTCF Binding Site, TF binding site, Open chromatin, Promoter and Enhancer information produced from ENCODE, Roadmap Epigenomics, and Blueprint projects [55] for GRCh37) and DNaseI Hypersensitivity Clusters in 125 cell types from ENCODE (V3) from the UCSC database [56] (GRCh37). Linkage disequilibrium information in these regional plots was produced using correlation *r*^2^ values for SNP pairs from PLINK, using genotyping data from 1000 Genomes Phase3 V5.

## Additional files

Additional file 1: Supplemental Data. Supplemental supporting figures (Figures S1–S10) and tables (Tables S1-S8). (PDF 4193 kb)

Additional file 2: Supplemental Table S5. Table containing Sherlock results. (PDF 68 kb)
