# Supplementary material for: Ensemble genomic analysis in human lung tissue identifies novel genes for chronic obstructive pulmonary disease
Source: Hum Genomics. 2018 Jan 15;12:1. doi: 10.1186/s40246-018-0132-z (PMC5769240; doi:10.1186/s40246-018-0132-z)
Supplement: Supplementary file 1 — Supplemental Data. Supplemental supporting figures (Figures S1–S10) and tables (Tables S1-S8). (PDF 4193 kb) [file 40246_2018_132_MOESM1_ESM.pdf]

Ensemble genomic analysis in human lung tissue identifies novel genes for chronic obstructive pulmonary disease

Jarrett D. Morrow, Michael H. Cho, John Platig, Xiaobo Zhou, Dawn L. DeMeo, Weiliang Qiu, Bartholome Celli, Nathaniel Marchetti, Gerard J. Criner, Raphael Bueno, George R. Washko, Kimberly Glass, John Quackenbush, Edwin K. Silverman, Craig P. Hersh

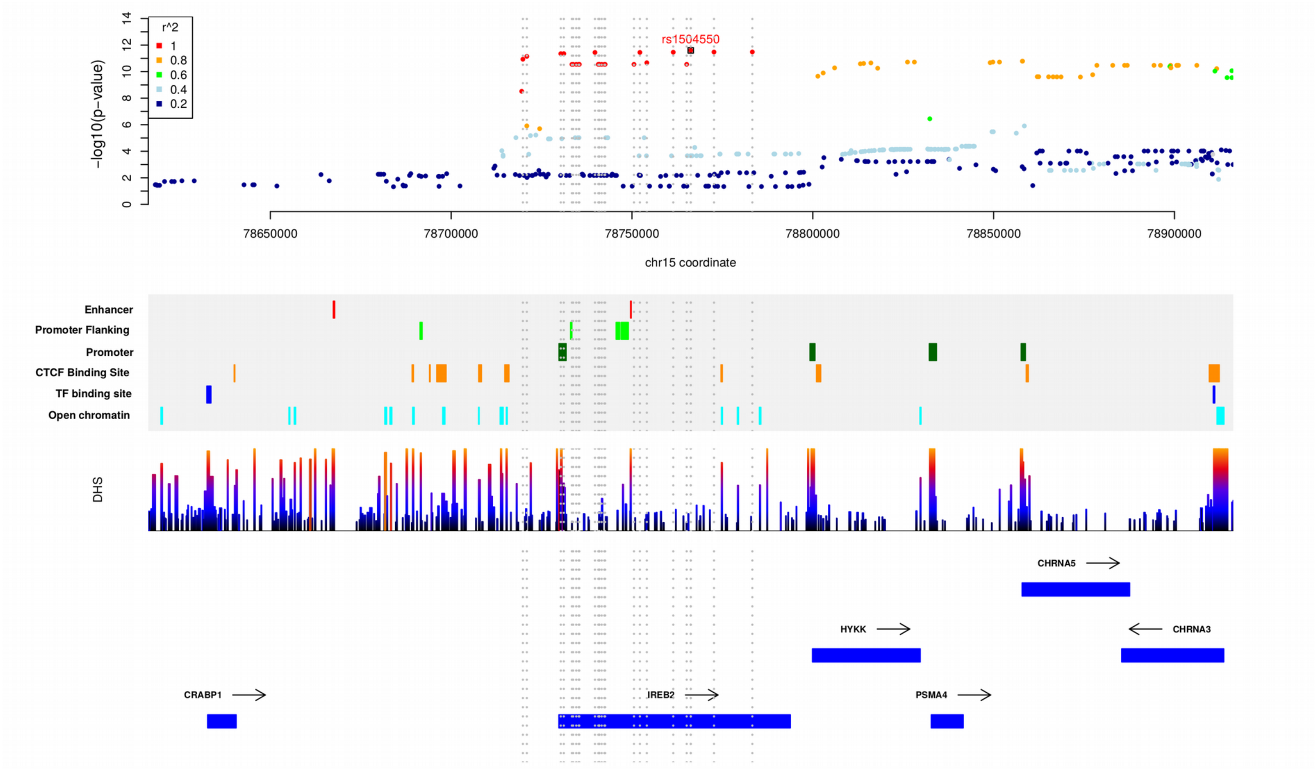

Figure S1. Regional genomic plot for rs1504550:*IREB2* locus with regulatory features and gene information; red and blue marker colors indicates higher and lower linkage disequilibrium (LD), respectively.

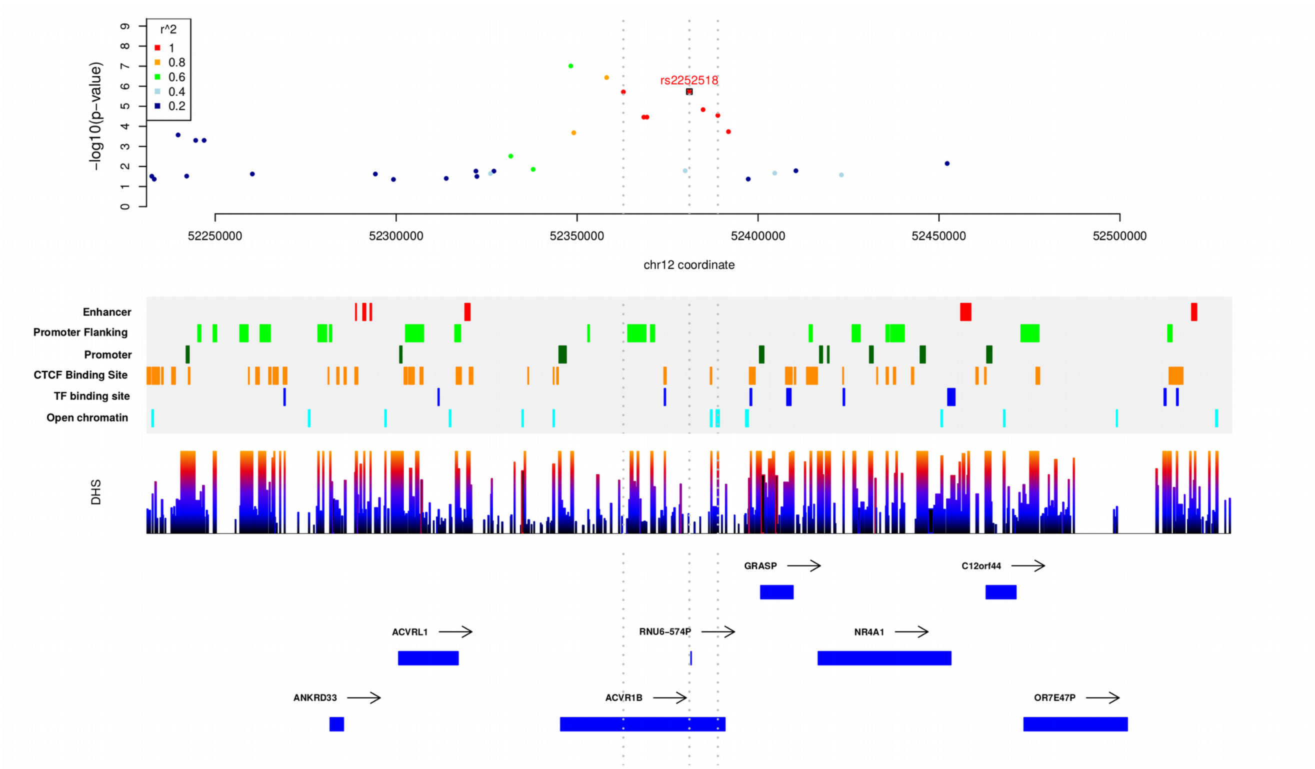

Figure S2. Regional genomic plot for rs2252518:*ACVR1B* locus with regulatory features and gene information; red and blue marker colors indicates higher and lower linkage disequilibrium (LD), respectively.

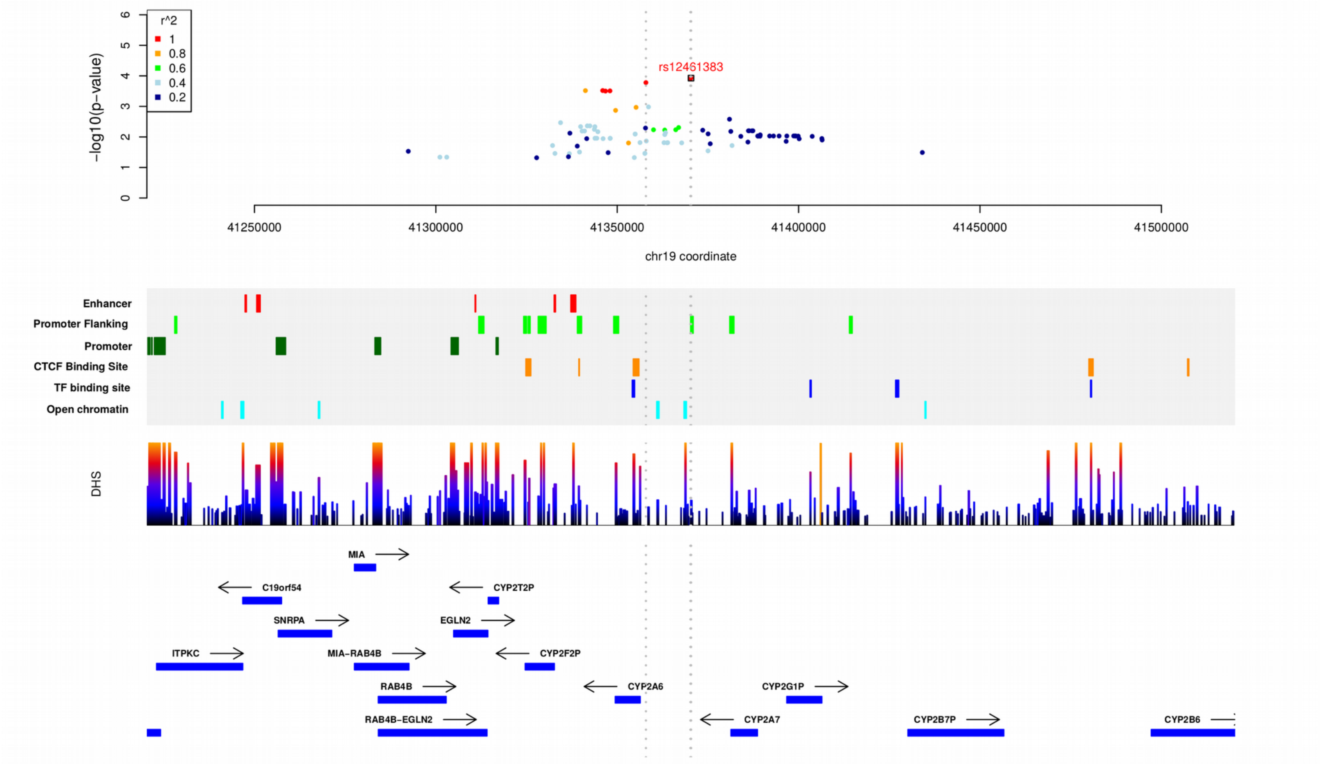

Figure S3. Regional genomic plot for rs12461383:*C19orf54* locus with regulatory features and gene information; red and blue marker colors indicates higher and lower linkage disequilibrium (LD), respectively.

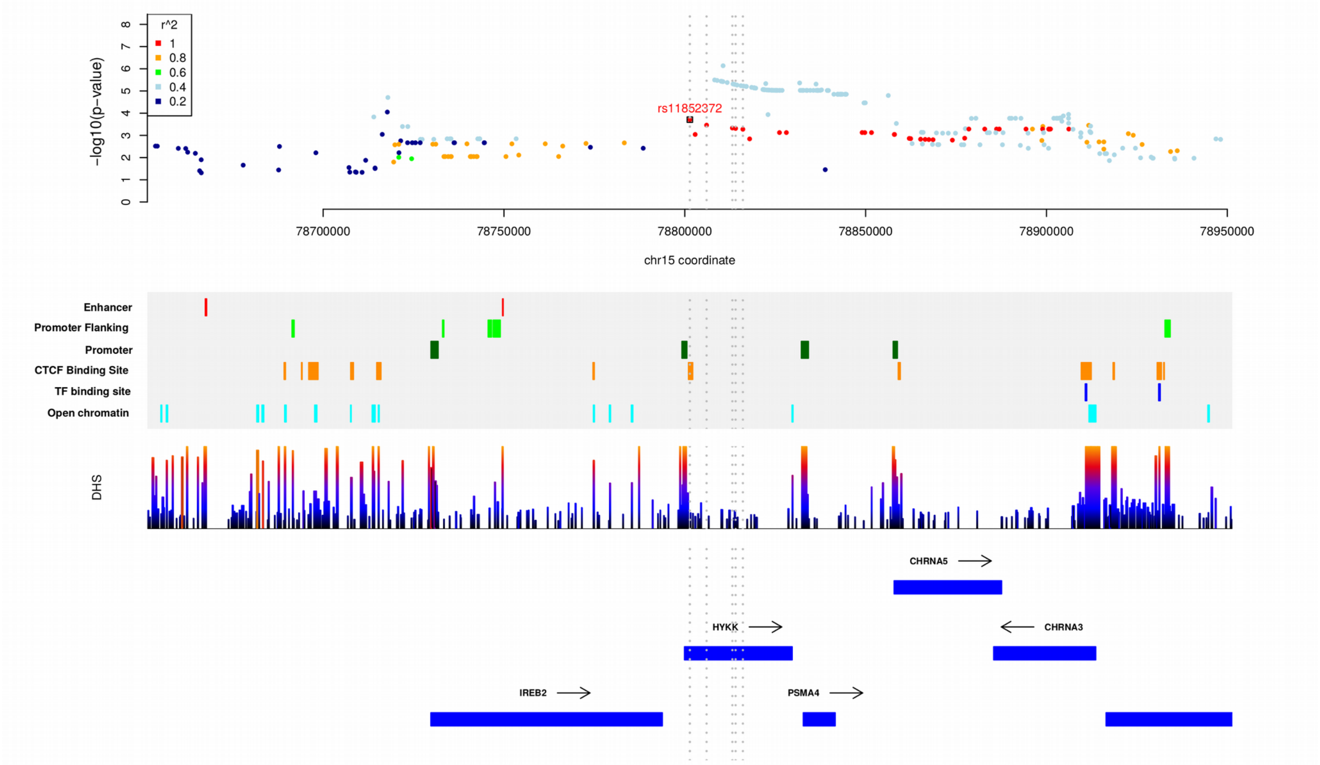

Figure S4. Regional genomic plot for rs11852372:*CHRNA5* locus with regulatory features and gene information; red and blue marker colors indicates higher and lower linkage disequilibrium (LD), respectively.

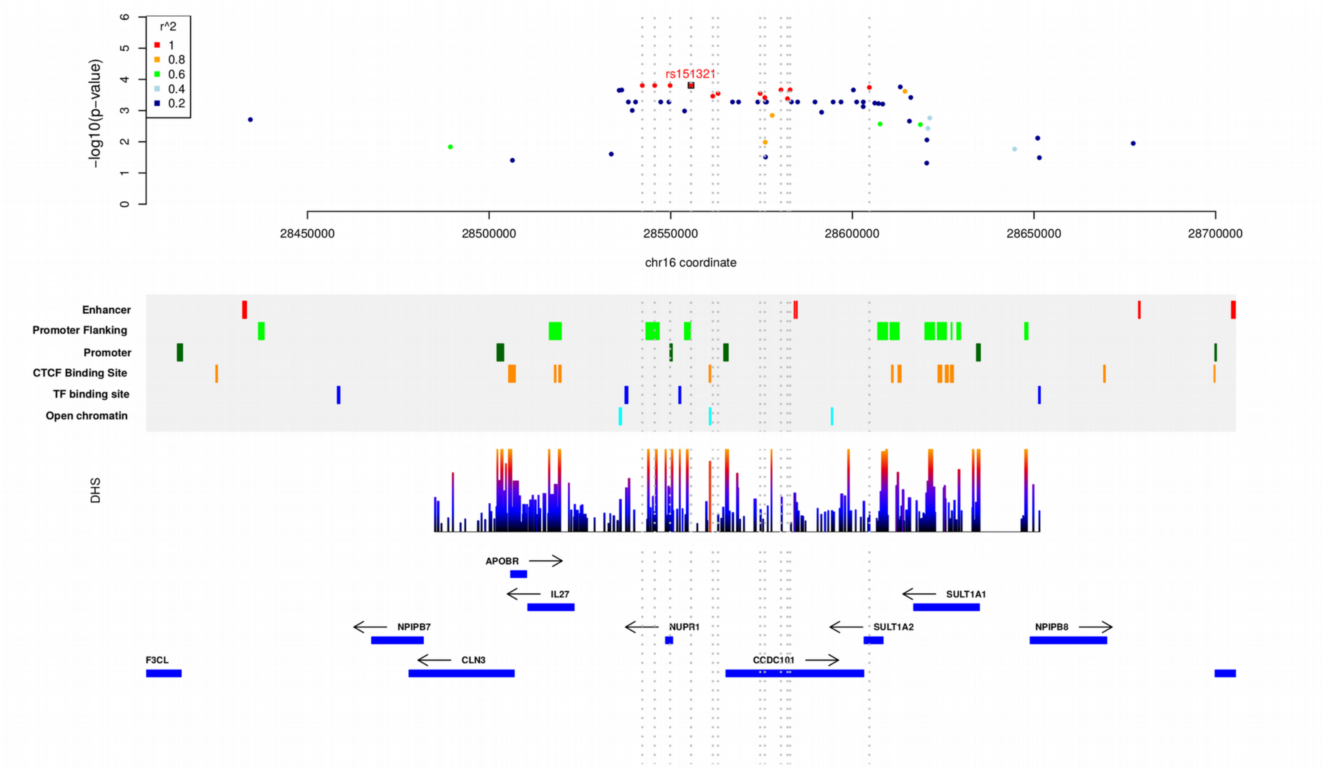

Figure S5. Regional genomic plot for rs151321:*SULT1A2* locus with regulatory features and gene information; red and blue marker colors indicates higher and lower linkage disequilibrium (LD), respectively.

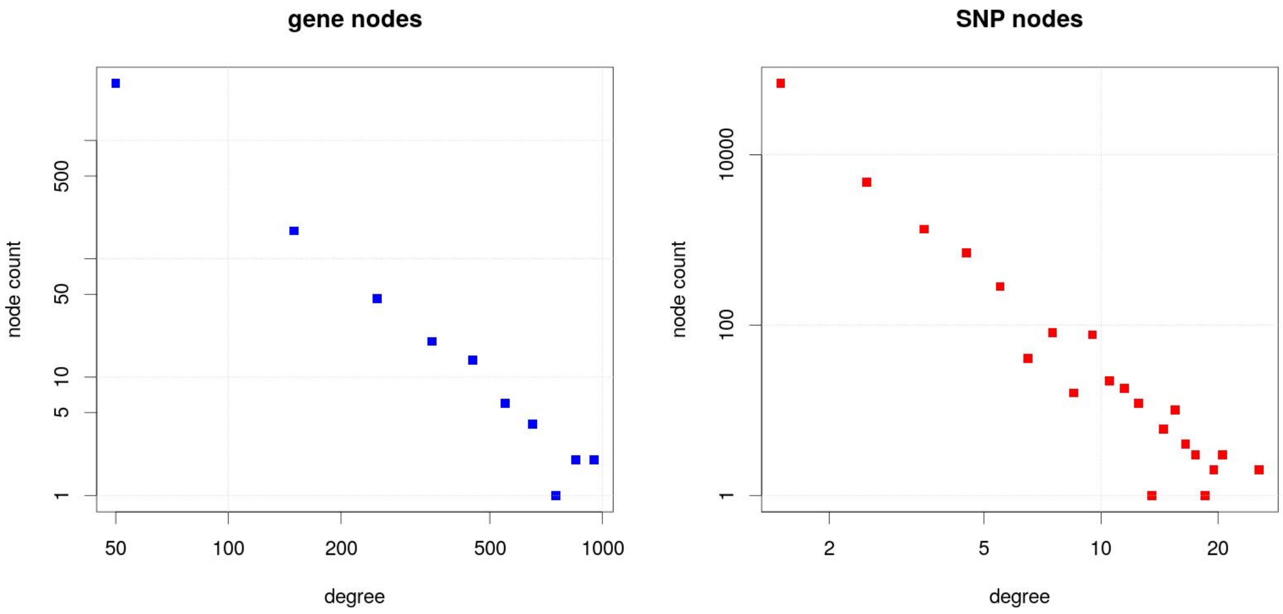

Figure S6. Degree distribution for the gene and SNP nodes

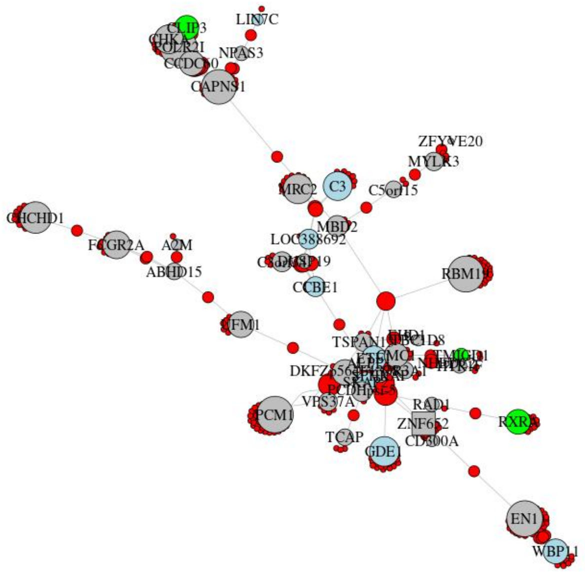

Figure S7. Community 218 containing the Sherlock gene *ZNF652*. Community genes are listed in Supplemental Table S6. (red = SNP, yellow = SNP with GWAS p<10^{-4}; square = Sherlock gene, gray = gene, green = gene with differentially methylated site (p<0.05 and effect>5%), light blue = gene with differentially expressed probe (p<0.05), cyan = gene with differentially methylated site and differentially expressed probe)

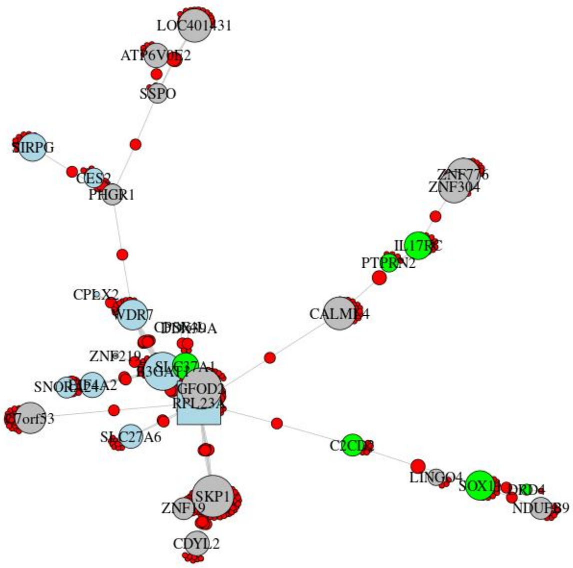

Figure S8. Community 223 containing the Sherlock gene *RPL23A* (sub-threshold SNP is hidden behind *RPL23A*). Community genes are listed in Supplemental Table S6. (red = SNP, yellow = SNP with GWAS p<10^{-4}; square = Sherlock gene, gray = gene, green = gene with differentially methylated site (p<0.05 and effect>5%), light blue = gene with differentially expressed probe (p<0.05), cyan = gene with differentially methylated site and differentially expressed probe)

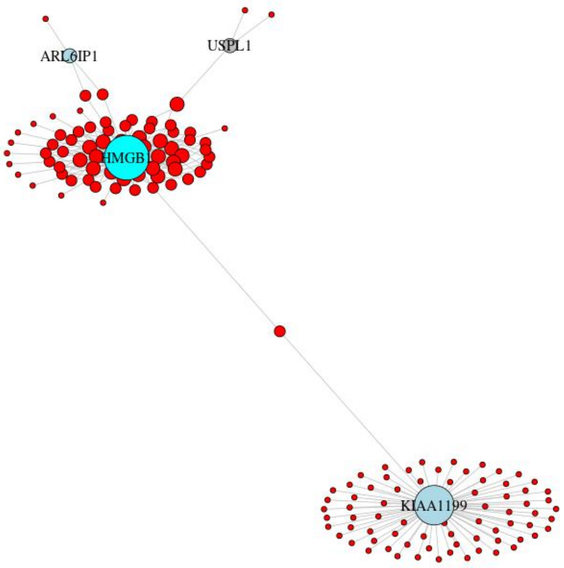

Figure S9. Community 98 containing the Sherlock gene *HMGB1*. Community genes are listed in Supplemental Table S6. (red = SNP, yellow = SNP with GWAS $p<10^{-4}$; square = Sherlock gene, gray = gene, green = gene with differentially methylated site ($p<0.05$ and effect>5%), light blue = gene with differentially expressed probe ($p<0.05$), cyan = gene with differentially methylated site and differentially expressed probe)

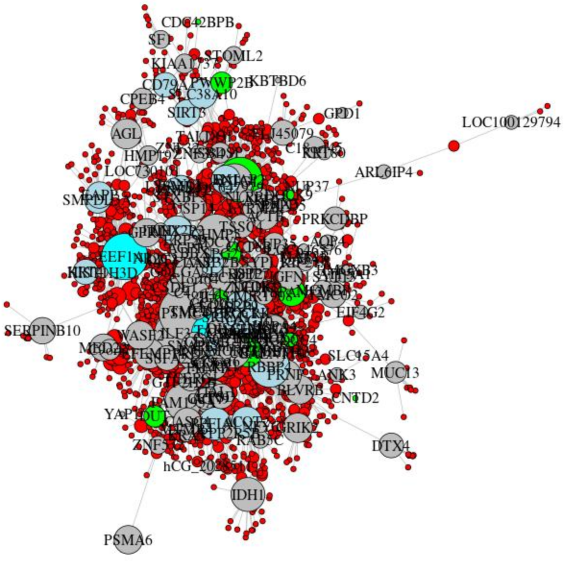

Figure S10. Community 135 containing the Sherlock gene *CD79A*. Community genes are listed in Supplemental Table S6. (red = SNP, yellow = SNP with GWAS $p<10^{-4}$; square = Sherlock gene, gray = gene, green = gene with differentially methylated site (p<0.05 and effect>5%), light blue = gene with differentially expressed probe (p<0.05), cyan = gene with differentially methylated site and differentially expressed probe)

Table S1. Demographics of study subjects with expression and genotyping data

**Demographics**	**COPD cases N=86**	**Control smokers N=31**	**P-value**
Age, years	64.1 (±6.4)	65.4 (±9.9)	0.50
Female Sex	45 (52.3%)	18 (58.1%)	0.68
White Race	100%	100%	NA
Former Smokers	100%	100%	NA
Smoking History, pack-years	65.2 (± 26.9)	36.9 (± 22.3)	<0.0001
Time since quitting, months	72.0 (± 64.2)	176.1 (± 144.9)	0.0006
FEV 1 % predicted	26.4 (± 9.9)	97.1 (± 10.6)	<0.0001
FEV 1 /FVC	0.32 (± 0.11)	0.79 (± 0.05)	<0.0001
Body Mass Index	25.5(±4.6)	27.8 (±4.5)	0.02
Abbreviations: FEV1=forced expiratory volume in 1 sec; FVC= forced vital capacity			

Table S2. Top 20 cis-expression quantitative trait locus results. Most significant eSNP for each gene is shown.

**probe ID**	**SNP**	**p-value**	**FDR q-value**	**Gene symbol**	**Chr**	**SNP to Gene distance (bases for hg19)**
ILMN_3236498	rs1060817	5.31E-46	6.28E-39	*LOC253039*	chr9	27793
ILMN_3298167	rs8066902	3.59E-44	1.82E-37	*ZSWIM7*	chr17	8518
ILMN_1753164	rs11834524	2.62E-41	1.34E-35	*IPO8*	chr12	26468
ILMN_1726647	rs10876864	1.56E-40	7.48E-35	*RPS26*	chr12	35761
ILMN_1697499	rs72851099	3.48E-39	1.49E-33	*HLA-DRB1*	chr6	13928
ILMN_1798177	rs10129479	3.10E-37	4.53E-32	*FNTB*	chr14	16401
ILMN_1719064	rs6663	3.29E-37	4.76E-32	*KCTD10*	chr12	14301
ILMN_1747935	rs113512099	3.07E-36	3.06E-31	*GOLGB1*	chr3	20812
ILMN_2345908	rs4931424	2.11E-35	1.25E-30	*DDX11*	chr12	12935
ILMN_2214278	rs755898	6.31E-35	3.46E-30	*ANKRD32*	chr5	11387
ILMN_2165753	rs9260193	1.34E-34	6.83E-30	*HLA-A*	chr6	497
ILMN_3250243	rs2551943	1.67E-34	8.42E-30	*METTL21A*	chr2	14232
ILMN_1782178	rs11120049	1.92E-34	9.15E-30	*LQK1*	chr1	2929
ILMN_1678974	rs2295716	2.40E-34	1.10E-29	*MRPL43*	chr10	1789
ILMN_1778488	rs150478765	2.78E-34	1.26E-29	*WDR41*	chr5	41198
ILMN_3286411	rs78789402	7.33E-34	3.15E-29	*C22orf41*	chr22	5810
ILMN_1719170	rs4717819	7.73E-34	3.31E-29	*WBSCR27*	chr7	17926
ILMN_2312606	rs2272347	9.99E-34	4.22E-29	*IRF5*	chr7	35372
ILMN_3251217	rs8060185	1.45E-33	5.76E-29	*PDXDC2*	chr16	52368
ILMN_2120982	rs2277448	1.54E-33	6.06E-29	*ALG11*	chr13	9603

Table S3. Top 20 trans-expression quantitative trait locus results. Most significant eSNP for each gene is shown.

probe ID	SNP	p-value	FDR q-value	Gene symbol	SNP Chromosome	Probe Chromosome
ILMN_3235221	rs7612	1.85E-38	2.71E-27	LOC644936	chr7	chr5
ILMN_2393693	rs2532340	1.14E-32	3.77E-23	LRRC37A4	chr17	chr17
ILMN_1724634	rs143761582	2.95E-26	4.36E-18	FLJ00326	chr4	chr8
ILMN_2118663	rs146271029	5.20E-26	7.55E-18	ERV3	chr7	chr7
ILMN_1675387	rs9271225	1.80E-21	2.02E-13	LIMS1	chr6	chr2
ILMN_3238751	rs2286990	6.05E-20	4.88E-12	hPMS6	chr7	chr7
ILMN_1825186	rs10772397	7.30E-20	5.81E-12	LOC338817	chr12	chr12
ILMN_3248654	rs2668668	3.98E-19	2.93E-11	C17orf69	chr17	chr17
ILMN_3308808	rs10876864	1.11E-17	5.84E-10	MIR130A	chr12	chr11
ILMN_1781236	rs2596492	5.40E-17	2.56E-09	LOC554223	chr6	chr6
ILMN_1767509	rs2760975	6.47E-17	3.03E-09	DEF8	chr6	chr16
ILMN_2415926	rs202223551	1.59E-16	6.98E-09	THOC3	chr5	chr5
ILMN_3308158	rs35159999	2.65E-16	1.13E-08	MIR330	chr4	chr19
ILMN_3278245	rs10222496	3.13E-16	1.32E-08	ZBTB20	chr3	chr3
ILMN_1745116	rs1203329	1.58E-15	6.19E-08	ABHD12	chr20	chr20
ILMN_1667452	rs141815353	3.91E-15	1.50E-07	LOC441208	chr7	chr7
ILMN_1722426	rs116178429	4.50E-15	1.72E-07	OR7D2	chr19	chr19
ILMN_3235044	rs71399704	5.77E-15	2.18E-07	LOC100288615	chr15	chr15
ILMN_1676528	rs35789010	5.92E-15	2.23E-07	BTN3A2	chr6	chr6
ILMN_3251217	rs7190039	1.42E-14	5.24E-07	PDXDC2	chr16	chr16

Table S4. Cis-eQTL results (FDR<5%) intersected with sub-threshold GWAS ($p<10^{-4}$). The most significant eSNP for each gene is shown.

**probe ID**	**SNP**	**eQTL p-value**	**eQTL FDR q-value**	**Gene symbol**	**Differential expression p-value**	**GWAS p-value**	**Chr**
ILMN_1726554	rs1504550	2.50E-12	3.27E-09	*IREB2*	0.119	6.35E-12	chr15
ILMN_1798308	rs1177287	1.60E-07	8.12E-05	*AHSA2*	0.499	1.51E-05	chr2
ILMN_1754501	rs1177292	1.54E-06	6.00E-04	*C2orf74*	0.292	7.83E-05	chr2
ILMN_2243308	rs2252518	1.85E-06	7.04E-04	*ACVR1B*	0.152	6.23E-05	chr12
ILMN_1738369	rs153106	2.30E-05	6.17E-03	*TUFM*	0.006	5.18E-05	chr16
ILMN_3238570	rs17707300	2.38E-05	6.34E-03	*EIF3CL*	0.644	5.16E-05	chr16
ILMN_2191192	rs2644899	2.79E-05	7.25E-03	*CYP2B7*	0.114	3.81E-05	chr19
ILMN_1767665	rs3111182	3.07E-05	7.85E-03	*GPX8*	0.116	8.00E-05	chr5
ILMN_1740319	rs61980585	8.13E-05	1.73E-02	*IFI27L2*	0.435	4.46E-05	chr14
ILMN_1729546	rs12461383	1.18E-04	2.34E-02	*C19orf54*	0.892	8.16E-06	chr19
ILMN_1798528	rs151321	1.55E-04	2.90E-02	*SULT1A2*	0.018	3.71E-05	chr16
ILMN_1657302	rs12447461	1.79E-04	3.23E-02	*SULT1A1*	0.563	9.08E-05	chr16
ILMN_1770044	rs11852372	1.96E-04	3.47E-02	*CHRNA5*	0.178	2.35E-11	chr15

Table S5. Top Sherlock results ($p < 10^{-3}$) including score for each expression quantitative trait locus

See additional file: Additional_File_2.pdf

Table S6. Genes within communities from Table 2

Comm ID	Sherlock or interactor gene(s)	Member genes
98	*HMGB1*	*ARL6IP1, HMGB1, KIAA1199, USPL1*
113	*CDH23*	*ALDH1A3, C14orf102, CDH23, CTDSPL2, FLJ34503, FOXRED2, GLO1, ID1, PSMC1, RBM43, ZCRB1, ZNF555*
135	*CD79A*	*ACN9, ACOT2, ACP1, ACTB, ACTG1, ADCY7, AGL, ANK3, ANP32B, ANX2P2, AQP4, ARF4, ARL6IP4, ATG4B, ATP2C1, ATP5A1, BAIAP2, BBX, BIRC2, BLVRB, BST2, C15orf44, C18orf45, C19orf6, C1orf64, C4orf41, C9orf7, CACYBP, CASC4, CCDC126, CCNC, CCS, CCT6B, CCT7, CD79A, CD79B, CDC42BPB, CHMP5, CLASP2, CNTD2, COG3, CPEB4, CRK, CSDE1, DISC1, DNAJA2, DOCK9, DPP7, DTX4, DUT, EAPP, EEF1A1, EFEMP1, EIF4G2, ERCC1, ERP29, FAM195A, FANCL, FLJ45079, FN1, GABPA, GALNT10, GATA3, GNG10, GOLGA2L1, GPD1, GPR1, GRIK2, GTF2H2B, hCG_2028511, HIST1H3D, HMGXB3, HMP19, HSPA4, IDH1, IGDCC4, IGFN1, IL6ST, ILF3, ING3, ITIH1, JAK1, KBTBD6, KIAA1737, KIF21A, KRAS, KRT4, KRT80, LHX3, LOC100129794, LOC646576, LOC647979, LOC730101, LYRM2, LYSMD3, MAT2B, MCMBP, MED22, MGMT, MIR1908, MOCS2, MORC3, MTMR4, MUC13, NCOR1, NFYC, NLRP1, NLRX1, NR2C2, NUP35, NUP37, OIT3, PAPD5, PEMT, PPFIA1, PPP2R5B, PRDX2, PRDX3, PRKCDBP, PRNP, PSD, PSMA6, PSMC4, PWWP2B, RAB5C, RBBP4, RNF8, SBDSP, SERPINB10, SF1, SIRT3, SLC15A4, SLC38A10, SMPDL3A, SNAPC5, SPG21, SRP9, SRRM1, SSFA2, STOML2, STXBP3, SYPL1, SYT13, TAF1L, TALDO1, TCERG1, TIAL1, TMCO2, TMED10, TMEM43, TSPAN12, TSSC4, TUBB, UBA3, USP14, WASF2, YAP1, ZDHHC2, ZNF32, ZNF384, ZNF442, ZNF572*
202	*CHRNA5*<br>*HNRNPAB*<br>*IREB2*<br>*PCBP2*	*C11orf80, CHRNA5, FBXO16, GGTLC1, HNRNPAB, IREB2, LIN54, LOC100286979, LOC283392, MAP6, PAQR6, PCBP2, RBM14, TARBP2, TECPR1, TLK1, ZNF114*
218	*ZNF652*	*A2M, ABHD15, ALPP, C3, C3orf34, C5orf15, CAPNS1, CCBE1, CCDC60, CD300A, CHCHD1, CHKA, CLIP3, CMC1, DKFZp564F1378, DUSP19, EHD1, EN1, ETF1, FCGR2A, GDE1, HTR1F, LIN7C, LOC388692, MBD2, MRC2, MYLK3, NHEDC2, NPAS3, OR3A1, PCDHpsi-5, PCM1, POLR2I, RAD1, RBM19, RXRA, SKAP2, SPHKAP, TBC1D8, TCAP, TMIGD1, TSPAN13, UFM1, VPS37A, WBP11, ZFYVE20, ZNF652*
222	*ACVR1B*	*ACVR1B, ADCY5, ADSSL1, ANKS1B, AP3D1, AQPEP, C19orf28, CHD4, CLEC4GP1, CPNE4, CRISPLD1, CSRNP1, DCLK1, DGKQ, DKKL1, EIF3C, FSCB, GMDS, HAND2, HSPB7, IDUA, ILK, INVS, IQGAP1, KIAA1429, LOC221442, LOC399959, LYNX1, MED13L, MFGE8, MPPED2, MYL9, MYOZ2, NCS1, NPR2, NTN1, NTRK3, PACS1, PDP1, PRDM16, PRDM6, PTPLA, RNF115, RNF138, RTN1, SCMH1, SCUBE3, SEMA4B, SGK196, SHC4, SHKBP1, SHROOM3, SLC10A7, SLC16A7, SLC25A4, SLMAP, SNORA1, SNRNP70, SRSF11, TCEB2, TCF3, TMC7, TRIP6, TRPT1, UCP3, VCL, ZNF609*
223	*RPL23A*	*ATP6V0E2, B3GAT1, C2CD2, C7orf53, CALML4, CDYL2, CES2, CPLX2, CPSF4L, DDX39A, DRD4, EIF4A2, GFOD2, IL17RC, LINGO4, LOC401431, NDUFB9, PHGR1, PTPRN2, RPL23A, SIRPG, SKP1, SLC27A6,*

		SLC37A1, SNORA24, SOX11, SSPO, WDR7, ZNF19, ZNF219, ZNF304, ZNF776
20	*WDR47*	*ACER2, AKD1, C14orf169, GPSM2, SMPD2, WDR47*
78	*CHIAP2*	*ATP5F1, C1orf88, CHI3L2, CHIA, DYRK3, FBN2, FOXM1, GABRB3, KCNN2, CHIAP2, LOC646324, LYN, PITX2, SGTA, SLC39A3, TICAM1, TIMM23, ZCWPW2*
131	*AHSA2*<br>*C2orf74*	*AHSA2, C2orf74, SLC33A1, XPO1*
161	*SMG6*	*ABLIM1, C17orf109, C17orf79, ENPP4, FBXO46, FLJ43663, GOLGA6L1, LOC728643, LTBP3, PAPD4, RPL11, RPS3A, SIGMAR1, SMG6, SRR, TCFL5, TMEM44, WDR23*
179	*DSP*	*DSP, EVI2B, FEM1A, FOXD4L1, OSR2, SF3B4, SPATA22*
181	*FSTL5*	*ATP6V0A4, CEP57, CLEC10A, CYCSP52, DKFZp686K04236, DPY19L2P2, DPY30, FAM92B, FSTL5, HMHB1, LOC728175, LYPLA2, MAF, MEF2D, MGC16075, MTMR14, NLRP7, PDPR, RAX2, RNF180, SNORD114-31, ZBTB2, ZNF668*
187	*SNRPB*	*GNA12, GTF2H4, HYAL4, NPDC1, POLR2J2, RNF5, RRM2B, SEPHS1, SIRT7, SNAPC2, SNRPB, TENC1, TUBB1, WASH2P*
210	*CTSH*	*AIF1, C10orf57, CTSH, GATSL3, GOLGB1, HCLS1, ILDR1, IQCB1, KEL, LSM11, STARD3, WDR5B*
249	*TRIM4*	*AZGP1, HIP1R, INTS6, KDM3B, MEPCE, PILRB, PODXL2, SFT2D2, TRIM4, TSC22D4, ZKSCAN1*

Table S7. Significant pathway analysis results (FDR q-value < 0.05) from ConsensusPathDB for the communities of interest

**Community ID (Sherlock or interactor gene)**	**Pathway name**	**Gene set size (number found among available expression data genes)**	**Overlap (percentage of available genes that overlap)**	**p-value**	**FDR q-value**	**pathway source**
222 (*ACVR1B*)	cGMP-PKG signaling pathway	168(145)	4 (2.8%)	0.0035	0.037	KEGG
	Bacterial invasion of epithelial cells	78(71)	3 (4.2%)	0.0036	0.037	KEGG
	Focal adhesion	201(178)	4 (2.2%)	0.0072	0.051	KEGG
135 (*CD79A*)	Folding of actin by CCT/TriC	10(10)	3 (30.0%)	0.00019	0.032	Reactome
	Regulation of actin dynamics for phagocytic cup formation	139(48)	5 (10.4%)	0.00025	0.032	Reactome
	RHO GTPases Activate WASPs and WAVEs	37(31)	4 (12.9%)	0.00047	0.037	Reactome
	Innate Immune System	1309(1000)	24 (2.4%)	0.00058	0.037	Reactome
	Fcgamma receptor (FCGR) dependent phagocytosis	157(64)	5 (7.8%)	0.00095	0.048	Reactome
	Immune System	1950(1519)	31 (2.0%)	0.0013	0.0497	Reactome
	CD22 mediated BCR regulation	71(5)	2 (40.0%)	0.00137	0.0497	Reactome

Table S8. Summary of genotype data quality control process

Subjects			Markers	
Genotyped subjects after initial standard QC	183		Genotyped markers after initial standard QC	2,556,097
Missingness > 5%	-0		Markers with technical issues	-3415
Relatedness (sample contamination/swap)	-6		Missingness > 1%	-149,608
Gender mismatch	-1		HWE (767 total flagged markers)	-709 unique
Duplicate sample	-1		Mapping issues and monomorphic	-498,967
Discordant genetic ancestry	-2		Markers passing QC – available for imputation	1,903,398
Control samples	-4		Markers after imputation with MAF>5% and info>0.3	6,347,053
Subject data available	169		Duplicate markers	-4
**Caucasian subjects with expression data**	**117**		Markers with MAF≤5% or info≤0.5 for subset of 117 subjects	-357,198
Female: 45 cases, 18 controls<br>Male: 41 cases, 13 controls			**Total markers included in analysis**	**5,989,851**
