# Supplementary material for: Ensemble genomic analysis in human lung tissue identifies novel genes for chronic obstructive pulmonary disease
Source: Hum Genomics. 2018 Jan 15;12:1. doi: 10.1186/s40246-018-0132-z (PMC5769240; doi:10.1186/s40246-018-0132-z)
Supplement: Supplementary file 2 — Supplemental Table S5. Table containing Sherlock results. (PDF 68 kb) [file 40246_2018_132_MOESM2_ESM.pdf]

Table S5. Top Sherlock results ($p < 10^{-3}$) including score for each expression quantitative trait locus

Sherlock Parameters

**# eQTL parameters**	
N_expr = 117	# sample size of the eQTL data (need to change for any eQTL dataset)
cis_expr_prior = 1.0E-3	# the prior of a cis-eSNP
trans_expr_prior = 5.0E-7	# the prior of a trans-eSNP (5.0E-5 default)
sigma_a_expr = 0.5	# the effect size prior of an expression QTL
**# GWAS parameter:**	**specified by the user except pheno_prior and sigma_a_pheno**
is_pheno_binary = 1	# whether the phenotypic trait is binary (1) or quantitative (0)
N_pheno = 12000	# sample size of the GWAS data
phi = 0.54	# the proportion of cases in the GWAS
K = 0.075	# the disease prevalance (COPD 24 million/318 million in US)
pheno_prior = 1.0E-3	# the prior of a phenotypic trait locus
sigma_a_pheno = 0.2	# the effect size prior of a phenotypic trait locus
**# other parameters:**	**no need to change**
cond_prior = 1.0	# the conditional prior given that the locus is a eSNP
cis_pheno_prior = 5e-4	# the prior of a phenotypic trait of a cis-SNP under H0
max_interval = 500000	# the interval size for defining a LD block

Sherlock Output

No. eqtlSNPs = 438536 No. gwasSNPs = 438536 No. commonSNPs = 438536
cisExprPrior = 0.001
transExprPrior = 5e-07
phenoPrior = 0.001
condPrior = 1

Gene Symbol	Total LBF Score	P-value					
MAPT	7.64821	6.91E-07					
**Gene Symbol**	**eSNP**	**chromosome**	**coordinates**	**cis or trans**	**eQTL p-value**	**GWAS p-value**	**LBF**
MAPT	rs7368400	1	158037320	trans	4.29E-07	9.96E-01	-3.09E-02
MAPT	rs963660	4	170995066	trans	1.03E-07	3.96E-01	-1.29E-02
MAPT	rs12261209	10	93389343	trans	8.68E-07	4.93E-01	-1.93E-03
MAPT	rs514960	13	51316115	trans	8.90E-07	3.80E-01	-5.73E-03
MAPT	rs55671319	17	43548424	cis	2.93E-13	6.33E-04	3.56E+00
MAPT	rs199508	17	44858838	trans	5.68E-12	1.90E-04	4.15E+00
MAPT	rs1877052	18	56754306	trans	3.40E-07	2.50E-01	-9.98E-03

Gene Symbol	Total LBF Score	P-value					
LRRC37A4	7.4567	6.91E-07					
**Gene Symbol**	**eSNP**	**chromosome**	**coordinates**	**cis or trans**	**eQTL p-value**	**GWAS p-value**	**LBF**
LRRC37A4	rs10495046	1	217417902	trans	2.08E-07	9.73E-01	-1.83E-03
LRRC37A4	rs72831485	2	127203633	trans	9.27E-07	5.67E-01	-3.50E-04
LRRC37A4	rs76094563	2	223616351	trans	6.36E-07	8.27E-01	-5.35E-03
LRRC37A4	rs4863838	4	131940633	trans	9.19E-07	4.99E-01	-1.73E-03
LRRC37A4	rs55671319	17	43548424	cis	2.17E-22	6.33E-04	3.28E+00
LRRC37A4	rs199508	17	44858838	trans	1.37E-19	1.90E-04	4.19E+00
LRRC37A4	rs8072731	17	50949615	trans	4.47E-07	5.49E-01	-2.67E-03
LRRC37A4	rs73612620	20	24759505	trans	8.00E-07	7.89E-02	1.07E-03

Gene Symbol	Total LBF Score	P-value					
C17orf69	7.38386	6.91E-07					
**Gene Symbol**	**eSNP**	**chromosome**	**coordinates**	**cis or trans**	**eQTL p-value**	**GWAS p-value**	**LBF**
C17orf69	rs34905331	3	72259486	trans	7.02E-07	5.49E-01	-1.66E-03
C17orf69	rs79346028	11	98087035	trans	5.28E-07	7.39E-02	-1.38E-03
C17orf69	rs55671319	17	43548424	cis	4.98E-18	6.33E-04	3.47E+00
C17orf69	rs199508	17	44858838	trans	5.08E-11	1.90E-04	3.92E+00

Gene Symbol	Total LBF Score	P-value					
IREB2	6.39628	6.91E-07					
**Gene Symbol**	**eSNP**	**chromosome**	**coordinates**	**cis or trans**	**eQTL p-value**	**GWAS p-value**	**LBF**
IREB2	rs186836436	2	59316963	trans	9.87E-07	9.31E-01	-3.26E-03
IREB2	rs1504550	15	78766250	cis	2.50E-12	6.35E-12	6.40E+00

Gene Symbol	Total LBF Score	P-value					
C19orf54	5.44542	5.53E-06					
**Gene Symbol**	**eSNP**	**chromosome**	**coordinates**	**cis or trans**	**eQTL p-value**	**GWAS p-value**	**LBF**
C19orf54	rs1031237	4	5213429	trans	5.73E-07	8.91E-01	-6.46E-03
C19orf54	rs17284079	5	82285005	trans	3.30E-07	1.08E-01	-1.59E-03
C19orf54	rs1739776	10	83923527	trans	2.17E-07	4.57E-02	2.47E-02
C19orf54	rs12461383	19	41370338	cis	1.18E-04	8.16E-06	5.43E+00

Gene Symbol	Total LBF Score	P-value
ACVR1B	5.40201	5.53E-06

Gene Symbol	eSNP	chromosome	coordinates	cis or trans	eQTL p-value	GWAS p-value	LBF
ACVR1B	rs12038544	1	100095361	trans	5.03E-07	4.20E-01	-6.21E-03
ACVR1B	rs2641530	12	52384811	cis	1.47E-05	6.25E-06	5.41E+00
ACVR1B	rs115215688	19	33639422	trans	7.25E-07	8.36E-01	-1.35E-03

Gene Symbol	Total LBF Score	P-value
EIF3CL	4.44554	1.94E-05

Gene Symbol	eSNP	chromosome	coordinates	cis or trans	eQTL p-value	GWAS p-value	LBF
EIF3CL	rs40837	16	28510845	cis	5.72E-05	8.67E-06	4.45E+00

Gene Symbol	Total LBF Score	P-value
TUFM	4.28685	2.49E-05

Gene Symbol	eSNP	chromosome	coordinates	cis or trans	eQTL p-value	GWAS p-value	LBF
TUFM	rs12446550	16	28543381	cis	2.30E-05	3.09E-05	4.29E+00

Gene Symbol	Total LBF Score	P-value
FAM13A	4.08652	3.60E-05

Gene Symbol	eSNP	chromosome	coordinates	cis or trans	eQTL p-value	GWAS p-value	LBF
FAM13A	rs2478801	1	70535186	trans	4.95E-07	9.36E-01	-4.12E-03
FAM13A	rs13110699	4	89815695	cis	4.02E-04	9.61E-06	4.04E+00
FAM13A	rs17473171	4	164474591	trans	2.56E-07	1.78E-01	-2.02E-03
FAM13A	rs112834401	4	188402490	trans	5.28E-07	2.53E-01	-4.01E-02
FAM13A	rs2233956	6	31081205	trans	9.20E-07	9.20E-01	-4.39E-03
FAM13A	rs630561	9	84414429	trans	4.23E-08	1.08E-02	1.12E-01
FAM13A	rs75717823	15	46936683	trans	2.98E-07	5.02E-01	-5.38E-03
FAM13A	rs11247059	15	99660664	trans	7.34E-07	8.80E-01	-7.44E-03

Gene Symbol	Total LBF Score	P-value
PCBP2	3.97202	4.43E-05

Gene Symbol	eSNP	chromosome	coordinates	cis or trans	eQTL p-value	GWAS p-value	LBF
PCBP2	rs60093138	1	239556578	trans	1.30E-07	1.89E-01	-1.77E-03
PCBP2	rs9973773	2	209943658	trans	5.06E-07	1.15E-01	-1.27E-03
PCBP2	rs2461796	4	149689983	trans	8.54E-07	5.17E-01	-6.81E-03
PCBP2	rs72754232	5	53523397	trans	4.07E-07	1.31E-01	2.31E-03
PCBP2	rs79255608	5	120876193	trans	3.42E-07	3.89E-01	-2.61E-03
PCBP2	rs57314710	8	58196051	trans	9.35E-07	9.98E-02	-2.58E-03
PCBP2	rs191856886	11	66756651	trans	9.48E-07	9.12E-01	-4.21E-04
PCBP2	rs67715155	12	54147863	cis	5.57E-04	1.28E-01	-1.17E-01
PCBP2	rs11630935	15	37562059	trans	4.59E-07	9.05E-01	-5.89E-03
PCBP2	rs2009746	15	78754102	trans	1.45E-08	1.27E-11	4.11E+00
PCBP2	rs11635742	15	86438207	trans	7.87E-07	3.82E-01	-5.82E-03
PCBP2	rs9900553	17	56008478	trans	7.99E-07	6.95E-01	-3.54E-04

Gene Symbol	Total LBF Score	P-value
CYP2B7	3.86813	5.67E-05

Gene Symbol	eSNP	chromosome	coordinates	cis or trans	eQTL p-value	GWAS p-value	LBF
CYP2B7	rs13202254	6	167718891	trans	8.56E-07	6.12E-01	-5.39E-03
CYP2B7	rs35799850	14	101156850	trans	9.04E-07	7.63E-01	-2.90E-03
CYP2B7	rs2644899	19	41302949	cis	2.79E-05	3.81E-05	3.88E+00

Gene Symbol	Total LBF Score	P-value
SULT1A1	3.81203	6.08E-05

Gene Symbol	eSNP	chromosome	coordinates	cis or trans	eQTL p-value	GWAS p-value	LBF
SULT1A1	rs79143008	8	89960108	trans	6.17E-07	2.81E-01	-1.14E-03
SULT1A1	rs10998683	10	53407216	trans	3.04E-07	1.22E-01	2.08E-03
SULT1A1	rs28449958	16	28528781	cis	1.24E-05	1.11E-04	3.81E+00

Gene Symbol	Total LBF Score	P-value
SULT1A2	3.80128	6.08E-05

Gene Symbol	eSNP	chromosome	coordinates	cis or trans	eQTL p-value	GWAS p-value	LBF
SULT1A2	rs231977	16	28542172	cis	1.56E-04	2.33E-05	3.80E+00

Gene Symbol	Total LBF Score	P-value
TIGD2	3.584	7.88E-05

Gene Symbol	eSNP	chromosome	coordinates	cis or trans	eQTL p-value	GWAS p-value	LBF
TIGD2	rs7692291	4	90055959	cis	4.30E-04	6.19E-06	3.60E+00
TIGD2	rs3109112	7	112095622	trans	8.06E-07	5.52E-01	-8.46E-03
TIGD2	rs1568484	8	119616401	trans	4.94E-07	2.72E-01	-2.38E-03
TIGD2	rs8112422	19	389615	trans	7.72E-07	3.82E-01	-8.78E-03

Gene Symbol	Total LBF Score	P-value
CHRNA5	3.37165	0.000105096

**Gene Symbol**	**eSNP**	**chromosome**	**coordinates**	**cis or trans**	**eQTL p-value**	**GWAS p-value**	**LBF**
CHRNA5	rs4727386	7	97688440	trans	7.94E-07	6.50E-01	-6.85E-03
CHRNA5	rs11852372	15	78801394	cis	1.96E-04	2.35E-11	3.38E+00
CHRNA5	rs68049718	16	83138643	trans	4.50E-07	3.73E-01	-4.17E-03
**Gene Symbol**	**Total LBF Score**	**P-value**					
BZRAP1	3.26582	0.000120307					
**Gene Symbol**	**eSNP**	**chromosome**	**coordinates**	**cis or trans**	**eQTL p-value**	**GWAS p-value**	**LBF**
BZRAP1	rs10269478	7	73922677	trans	5.73E-07	6.79E-02	3.83E-03
BZRAP1	rs10096068	8	57864597	trans	6.72E-08	1.38E-04	3.09E+00
BZRAP1	rs78160339	12	103735918	trans	1.82E-07	3.03E-01	-3.61E-03
BZRAP1	rs62084580	17	56390249	cis	1.19E-04	1.99E-02	1.78E-01
**Gene Symbol**	**Total LBF Score**	**P-value**					
GPX8	3.24936	0.000120307					
**Gene Symbol**	**eSNP**	**chromosome**	**coordinates**	**cis or trans**	**eQTL p-value**	**GWAS p-value**	**LBF**
GPX8	rs71449001	2	18939286	trans	6.23E-07	4.41E-01	-8.74E-04
GPX8	rs4318611	4	159732703	trans	1.04E-07	3.60E-02	8.25E-02
GPX8	rs2069187	5	54398049	cis	3.19E-05	2.17E-05	3.17E+00
**Gene Symbol**	**Total LBF Score**	**P-value**					
TEKT3	3.18918	0.00013137					
**Gene Symbol**	**eSNP**	**chromosome**	**coordinates**	**cis or trans**	**eQTL p-value**	**GWAS p-value**	**LBF**
TEKT3	rs1555378	6	124637400	trans	8.28E-07	6.17E-01	-1.82E-03
TEKT3	rs2168999	17	15237488	cis	4.80E-09	1.31E-04	3.19E+00
**Gene Symbol**	**Total LBF Score**	**P-value**					
SNRPB	3.06387	0.000163175					
**Gene Symbol**	**eSNP**	**chromosome**	**coordinates**	**cis or trans**	**eQTL p-value**	**GWAS p-value**	**LBF**
SNRPB	rs6427668	1	162287125	trans	2.46E-07	3.92E-01	-3.83E-04
SNRPB	rs34510008	2	103574174	trans	4.11E-08	2.71E-01	-5.12E-02
SNRPB	rs10039101	5	9689026	trans	4.91E-07	4.72E-01	-8.59E-03
SNRPB	rs62493121	8	13636171	trans	2.40E-07	2.94E-01	-2.09E-03
SNRPB	rs146137095	8	31811687	trans	1.43E-10	1.49E-03	3.13E+00
SNRPB	rs78773284	8	85112032	trans	3.91E-07	2.13E-01	-6.54E-04
SNRPB	rs66596168	8	135916183	trans	8.65E-07	3.94E-01	-2.75E-03
SNRPB	rs76421149	12	117737646	trans	5.12E-07	5.14E-01	-1.17E-04
SNRPB	rs138851638	19	57192149	trans	2.38E-07	7.49E-01	-1.48E-04
**Gene Symbol**	**Total LBF Score**	**P-value**					
ZNF652	3.0261	0.000168706					
**Gene Symbol**	**eSNP**	**chromosome**	**coordinates**	**cis or trans**	**eQTL p-value**	**GWAS p-value**	**LBF**
ZNF652	rs79808400	2	119350062	trans	8.72E-07	1.48E-01	8.54E-04
ZNF652	rs13062703	3	196070280	trans	3.43E-07	7.01E-01	-1.29E-02
ZNF652	rs79947894	8	17576894	trans	1.10E-07	5.52E-02	6.53E-03
ZNF652	rs8075581	17	47037962	cis	4.78E-05	3.20E-04	3.03E+00
**Gene Symbol**	**Total LBF Score**	**P-value**					
AHSA2	2.8564	0.00021434					
**Gene Symbol**	**eSNP**	**chromosome**	**coordinates**	**cis or trans**	**eQTL p-value**	**GWAS p-value**	**LBF**
AHSA2	rs1186708	2	61673706	cis	2.62E-07	1.12E-05	2.86E+00
AHSA2	rs6537001	4	141555651	trans	5.02E-07	8.71E-01	-2.39E-03
**Gene Symbol**	**Total LBF Score**	**P-value**					
CDH23	2.82403	0.000221254					
**Gene Symbol**	**eSNP**	**chromosome**	**coordinates**	**cis or trans**	**eQTL p-value**	**GWAS p-value**	**LBF**
CDH23	rs1221518	2	164193154	trans	5.98E-07	6.84E-01	-6.83E-03
CDH23	rs146798790	3	67200754	trans	1.95E-07	8.97E-01	-2.65E-04
CDH23	rs79241380	4	22521218	trans	4.60E-07	3.98E-01	-4.15E-03
CDH23	rs2746171	6	38719531	trans	1.04E-07	6.03E-01	-4.09E-02
CDH23	rs4747180	10	73412757	cis	5.45E-07	2.57E-04	2.88E+00
CDH23	rs11064196	12	6556686	trans	7.50E-07	7.06E-01	-7.14E-03
**Gene Symbol**	**Total LBF Score**	**P-value**					
NOP2	2.68909	0.000298693					
**Gene Symbol**	**eSNP**	**chromosome**	**coordinates**	**cis or trans**	**eQTL p-value**	**GWAS p-value**	**LBF**
NOP2	rs3020326	6	152295185	trans	9.87E-07	2.48E-01	-4.53E-03
NOP2	rs7083616	10	2633065	trans	1.06E-08	9.20E-04	2.69E+00
**Gene Symbol**	**Total LBF Score**	**P-value**					
AASDH	2.67671	0.000305607					
**Gene Symbol**	**eSNP**	**chromosome**	**coordinates**	**cis or trans**	**eQTL p-value**	**GWAS p-value**	**LBF**
AASDH	rs13135046	4	57274617	cis	2.59E-07	2.39E-04	2.68E+00
**Gene Symbol**	**Total LBF Score**	**P-value**					

DAGLA	2.67663	0.000305607

Gene Symbol	eSNP	chromosome	coordinates	cis or trans	eQTL p-value	GWAS p-value	LBF
DAGLA	rs2668193	3	183936680	trans	2.41E-07	4.78E-05	2.71E+00
DAGLA	rs76943104	4	40461091	trans	4.49E-07	2.22E-01	-3.01E-03
DAGLA	rs11954573	5	76035067	trans	7.41E-07	1.77E-01	-3.35E-03
DAGLA	rs35486442	11	61816314	cis	5.97E-04	7.50E-01	-2.49E-02

Gene Symbol	Total LBF Score	P-value
IFI27L2	2.65426	0.000323584

Gene Symbol	eSNP	chromosome	coordinates	cis or trans	eQTL p-value	GWAS p-value	LBF
IFI27L2	rs7001816	8	142039892	trans	6.29E-07	1.08E-01	-1.60E-03
IFI27L2	rs61980585	14	94768833	cis	8.13E-05	4.46E-05	2.66E+00

Gene Symbol	Total LBF Score	P-value
APIP	2.60025	0.000348475

Gene Symbol	eSNP	chromosome	coordinates	cis or trans	eQTL p-value	GWAS p-value	LBF
APIP	rs6692356	1	200455322	trans	6.23E-07	9.45E-01	-1.31E-03
APIP	rs13018026	2	106675915	trans	9.09E-07	7.17E-01	-2.74E-03
APIP	rs78558198	3	30179099	trans	6.56E-07	4.65E-01	-1.98E-03
APIP	rs112109124	4	44349675	trans	7.13E-07	2.00E-01	-4.67E-04
APIP	rs7703800	5	110203417	trans	2.38E-07	1.11E-01	-7.70E-03
APIP	rs4744244	9	96242455	trans	9.20E-07	6.88E-01	-1.50E-03
APIP	rs7119958	11	34956616	cis	3.49E-09	5.85E-04	2.66E+00
APIP	rs17174000	11	43399401	trans	1.77E-07	7.44E-01	-1.61E-03
APIP	rs11218046	11	98887130	trans	5.88E-07	8.24E-01	-2.66E-03
APIP	rs1678953	12	122385213	trans	4.58E-07	6.01E-01	-8.71E-03
APIP	rs11623790	14	48732152	trans	2.48E-07	7.36E-01	-5.15E-03
APIP	rs7342923	17	8723009	trans	8.06E-08	8.69E-01	-2.03E-02
APIP	rs4803630	19	43951083	trans	6.39E-07	6.03E-01	-2.70E-03
APIP	rs55797396	20	43457618	trans	8.23E-07	3.18E-01	-2.91E-03

Gene Symbol	Total LBF Score	P-value
AXIN2	2.59432	0.00035539

Gene Symbol	eSNP	chromosome	coordinates	cis or trans	eQTL p-value	GWAS p-value	LBF
AXIN2	rs35918067	4	154149894	trans	9.09E-07	2.70E-02	1.79E-02
AXIN2	rs12947336	17	63832621	cis	5.88E-05	1.18E-03	2.58E+00

Gene Symbol	Total LBF Score	P-value
WDR47	2.49124	0.000420383

Gene Symbol	eSNP	chromosome	coordinates	cis or trans	eQTL p-value	GWAS p-value	LBF
WDR47	rs139317900	1	109582123	cis	1.09E-18	3.24E-04	2.52E+00
WDR47	rs9849237	3	2675189	trans	2.36E-07	5.99E-01	-1.48E-02
WDR47	rs74855156	9	4460023	trans	6.35E-07	6.25E-01	-4.07E-03
WDR47	rs111929430	9	36447046	trans	8.10E-07	8.37E-01	-1.51E-03
WDR47	rs756924	11	2323029	trans	5.71E-07	8.12E-01	-2.42E-03
WDR47	rs201861	11	32616834	trans	5.91E-07	5.17E-01	-6.80E-03

Gene Symbol	Total LBF Score	P-value
C4orf33	2.41012	0.000474314

Gene Symbol	eSNP	chromosome	coordinates	cis or trans	eQTL p-value	GWAS p-value	LBF
C4orf33	rs1596963	4	129912966	cis	3.28E-08	5.18E-04	2.41E+00

Gene Symbol	Total LBF Score	P-value
HNRNPAB	2.33652	0.000522713

Gene Symbol	eSNP	chromosome	coordinates	cis or trans	eQTL p-value	GWAS p-value	LBF
HNRNPAB	rs1168576	10	27908972	trans	2.74E-07	4.56E-01	-1.34E-02
HNRNPAB	rs72738736	15	78765122	trans	5.24E-07	1.73E-10	2.35E+00

Gene Symbol	Total LBF Score	P-value
GFPT1	2.33066	0.000524096

Gene Symbol	eSNP	chromosome	coordinates	cis or trans	eQTL p-value	GWAS p-value	LBF
GFPT1	rs111600359	2	36867639	trans	8.75E-07	2.44E-02	2.26E-02
GFPT1	rs11884868	2	69645765	cis	6.74E-09	2.70E-04	2.31E+00
GFPT1	rs57095742	3	157169017	trans	9.80E-07	2.59E-01	-3.16E-03

Gene Symbol	Total LBF Score	P-value
LOC644172	2.31936	0.000529627

Gene Symbol	eSNP	chromosome	coordinates	cis or trans	eQTL p-value	GWAS p-value	LBF
LOC644172	rs16940665	17	43907896	cis	4.73E-05	7.00E-04	2.32E+00

Gene Symbol	Total LBF Score	P-value
SNORD25	2.24523	0.000586324

Gene Symbol	eSNP	chromosome	coordinates	cis or trans	eQTL p-value	GWAS p-value	LBF
SNORD25	rs493851	11	62617311	cis	1.02E-08	7.11E-04	2.25E+00

Gene Symbol	Total LBF Score	P-value					
PPAT	2.23273	0.000605683					
**Gene Symbol**	**eSNP**	**chromosome**	**coordinates**	**cis or trans**	**eQTL p-value**	**GWAS p-value**	**LBF**
PPAT	rs13135046	4	57274617	cis	2.79E-05	2.39E-04	2.25E+00
PPAT	rs10938017	4	73246388	trans	5.65E-07	3.06E-01	-1.10E-02
PPAT	rs6482917	10	129248986	trans	3.62E-07	3.79E-01	-6.09E-03
PPAT	rs10848029	12	130665452	trans	1.06E-07	1.80E-01	-8.59E-04

Gene Symbol	Total LBF Score	P-value					
FBRSL1	2.22667	0.000608449					
**Gene Symbol**	**eSNP**	**chromosome**	**coordinates**	**cis or trans**	**eQTL p-value**	**GWAS p-value**	**LBF**
FBRSL1	rs10870469	12	133142686	cis	1.94E-04	1.28E-04	2.23E+00
FBRSL1	rs9557803	13	102749047	trans	9.25E-07	4.82E-01	-4.40E-03

Gene Symbol	Total LBF Score	P-value					
FSTL5	2.22195	0.000613981					
**Gene Symbol**	**eSNP**	**chromosome**	**coordinates**	**cis or trans**	**eQTL p-value**	**GWAS p-value**	**LBF**
FSTL5	rs12409038	1	4095750	trans	6.85E-07	1.06E-01	2.12E-03
FSTL5	rs10927551	1	15123809	trans	2.71E-07	8.88E-01	-1.01E-02
FSTL5	rs111594663	2	32510811	trans	5.77E-08	2.39E-01	-9.10E-03
FSTL5	rs35834092	4	38998449	trans	9.81E-07	4.15E-01	-3.01E-01
FSTL5	rs11100323	4	161890161	cis	6.60E-04	3.83E-02	1.84E-02
FSTL5	rs187403598	4	180684799	trans	9.31E-07	3.50E-02	8.11E-03
FSTL5	rs17501392	5	16707837	trans	3.57E-08	5.82E-01	-4.28E-02
FSTL5	rs2227469	5	148756728	trans	1.12E-07	5.47E-01	-9.66E-03
FSTL5	rs62386869	5	158133674	trans	9.56E-09	2.28E-01	-2.65E-02
FSTL5	rs56849581	6	36195966	trans	6.59E-07	5.73E-01	-2.86E-03
FSTL5	rs2518204	6	102318647	trans	4.62E-08	9.44E-04	1.21E+00
FSTL5	rs4461743	6	155491338	trans	1.97E-07	6.36E-01	-3.48E-03
FSTL5	rs73670309	7	1065947	trans	1.59E-07	2.90E-02	4.57E-02
FSTL5	rs4728215	7	130960827	trans	8.91E-07	1.87E-01	-2.67E-03
FSTL5	rs73183367	8	3543663	trans	2.92E-08	1.61E-02	2.93E-02
FSTL5	rs11521323	9	19392741	trans	8.33E-09	7.38E-01	-1.26E-01
FSTL5	rs10823609	10	72504087	trans	1.64E-07	9.75E-01	-8.26E-03
FSTL5	rs1621857	10	118557507	trans	3.13E-08	2.11E-01	-1.58E-03
FSTL5	rs143460354	11	17756788	trans	3.27E-07	8.36E-01	-4.70E-03
FSTL5	rs79915089	11	80018030	trans	1.78E-07	6.70E-01	-7.07E-03
FSTL5	rs7115104	11	95864253	trans	7.04E-07	8.91E-03	3.28E-02
FSTL5	rs12883159	14	60142500	trans	3.95E-07	2.94E-01	-8.29E-04
FSTL5	rs72700362	14	94447966	trans	9.81E-07	4.32E-01	-4.45E-04
FSTL5	rs56305315	16	75496758	trans	5.86E-07	3.15E-02	1.78E-02
FSTL5	rs72859855	17	68847018	trans	4.58E-11	1.61E-01	1.25E-01
FSTL5	rs117501783	18	8120494	trans	9.97E-12	7.75E-03	1.31E+00
FSTL5	rs35836050	21	43727291	trans	1.83E-07	7.50E-01	-7.69E-03
FSTL5	rs34120785	22	40664083	trans	5.04E-08	8.36E-01	-1.78E-02

Gene Symbol	Total LBF Score	P-value					
SMG6	2.18245	0.000644403					
**Gene Symbol**	**eSNP**	**chromosome**	**coordinates**	**cis or trans**	**eQTL p-value**	**GWAS p-value**	**LBF**
SMG6	rs7587916	2	234586814	trans	2.87E-08	2.26E-04	2.21E+00
SMG6	rs1115498	11	22435434	trans	7.24E-07	8.87E-01	-6.20E-03
SMG6	rs2957924	17	1953037	cis	6.89E-04	4.79E-01	-2.52E-02

Gene Symbol	Total LBF Score	P-value					
LOC149620	2.08524	0.000757796					
**Gene Symbol**	**eSNP**	**chromosome**	**coordinates**	**cis or trans**	**eQTL p-value**	**GWAS p-value**	**LBF**
LOC149620	rs10857870	1	111829220	cis	1.38E-11	5.19E-04	2.09E+00
LOC149620	rs11569266	3	141681078	trans	9.79E-07	6.10E-02	3.72E-03
LOC149620	rs13301360	9	31222719	trans	9.57E-07	6.82E-01	-9.27E-03
LOC149620	rs653359	9	79334707	trans	7.49E-08	2.14E-01	-2.91E-03

Gene Symbol	Total LBF Score	P-value					
RPL23A	2.0673	0.000777156					
**Gene Symbol**	**eSNP**	**chromosome**	**coordinates**	**cis or trans**	**eQTL p-value**	**GWAS p-value**	**LBF**
RPL23A	rs11677383	2	39495031	trans	9.34E-07	8.10E-01	-5.35E-03
RPL23A	rs35694566	2	62674238	trans	2.13E-07	4.19E-01	-7.98E-03
RPL23A	rs62275028	3	89543067	trans	2.62E-07	9.15E-01	-1.04E-02
RPL23A	rs73842249	4	89746849	trans	7.94E-07	6.26E-06	2.16E+00
RPL23A	rs17167478	5	133703592	trans	9.71E-07	3.37E-01	-3.47E-03
RPL23A	rs75289391	7	112420863	trans	2.56E-07	3.60E-02	-1.72E-04
RPL23A	rs56332133	8	21928635	trans	7.48E-07	4.28E-01	-6.68E-03
RPL23A	rs6476249	9	30950959	trans	7.53E-07	9.60E-01	-7.35E-03
RPL23A	rs62563765	9	74631464	trans	9.72E-07	8.33E-01	-2.04E-03
RPL23A	rs561774	11	94340838	trans	3.77E-07	5.14E-01	-3.18E-03
RPL23A	rs7136795	12	115813607	trans	6.16E-07	3.46E-01	-7.05E-03

RPL23A	rs1834426	14	70676661	trans	7.84E-07	8.09E-01	-7.59E-03
RPL23A	rs183051	17	26566523	cis	9.68E-04	9.24E-01	-2.52E-02
RPL23A	rs76846981	17	27202885	cis	9.06E-04	7.36E-02	-1.97E-03
RPL23A	rs57082072	20	3828150	trans	8.70E-07	8.12E-01	-1.48E-03

Gene Symbol	Total LBF Score	P-value
C2orf74	2.06035	0.000785453

Gene Symbol	eSNP	chromosome	coordinates	cis or trans	eQTL p-value	GWAS p-value	LBF
C2orf74	rs1177284	2	61349446	cis	1.80E-06	1.50E-05	2.08E+00
C2orf74	rs11531480	7	16771045	trans	6.94E-07	9.42E-01	-7.18E-03
C2orf74	rs79110990	9	18148302	trans	4.51E-07	4.37E-01	-8.78E-03

Gene Symbol	Total LBF Score	P-value
CTSH	2.03935	0.000817258

Gene Symbol	eSNP	chromosome	coordinates	cis or trans	eQTL p-value	GWAS p-value	LBF
CTSH	rs75640422	3	121457244	trans	8.26E-07	4.52E-01	-3.30E-03
CTSH	rs10440769	5	118437042	trans	2.50E-07	1.23E-01	1.79E-03
CTSH	rs28360647	6	128825695	trans	7.37E-07	2.37E-01	-1.29E-04
CTSH	rs6944334	7	50018441	trans	6.20E-08	7.58E-01	-1.04E-02
CTSH	rs117688148	14	74749376	trans	6.98E-07	7.33E-01	-3.64E-04
CTSH	rs62002013	15	25362079	trans	7.36E-08	1.05E-01	1.46E-02
CTSH	rs11633178	15	78944538	cis	7.16E-04	7.05E-08	2.04E+00
CTSH	rs4796412	17	7280269	trans	7.92E-07	3.99E-01	-2.57E-03

Gene Symbol	Total LBF Score	P-value
UBE2J1	2.03398	0.000826938

Gene Symbol	eSNP	chromosome	coordinates	cis or trans	eQTL p-value	GWAS p-value	LBF
UBE2J1	rs6788625	3	71929675	trans	7.91E-07	4.97E-01	-5.08E-03
UBE2J1	rs7764923	6	90022921	cis	8.66E-04	2.97E-04	2.04E+00

Gene Symbol	Total LBF Score	P-value
AEN	2.01443	0.000850446

Gene Symbol	eSNP	chromosome	coordinates	cis or trans	eQTL p-value	GWAS p-value	LBF
AEN	rs16860416	2	173316788	trans	2.17E-08	6.54E-01	-5.05E-02
AEN	rs9837963	3	61956001	trans	8.44E-07	1.87E-01	-3.40E-03
AEN	rs11739886	5	5326788	trans	1.79E-07	1.96E-04	2.07E+00
AEN	rs72816708	5	173541473	trans	9.64E-07	3.59E-01	-1.74E-03

Gene Symbol	Total LBF Score	P-value
CUL1	1.99742	0.000887783

Gene Symbol	eSNP	chromosome	coordinates	cis or trans	eQTL p-value	GWAS p-value	LBF
CUL1	rs9876870	3	156167184	trans	1.22E-07	8.80E-01	-3.69E-02
CUL1	rs1177950	7	148056135	cis	8.52E-04	2.53E-04	2.04E+00
CUL1	rs2049135	15	37585974	trans	6.74E-07	5.79E-01	-7.66E-03

Gene Symbol	Total LBF Score	P-value
DSP	1.99613	0.000890548

Gene Symbol	eSNP	chromosome	coordinates	cis or trans	eQTL p-value	GWAS p-value	LBF
DSP	rs2076295	6	7563232	cis	2.25E-08	3.89E-04	2.00E+00
DSP	rs77720963	10	29935759	trans	7.76E-07	3.18E-01	-3.26E-04
DSP	rs12425043	12	114864618	trans	2.13E-07	6.49E-02	-1.37E-03

Gene Symbol	Total LBF Score	P-value
MYCN	1.97048	0.000932033

Gene Symbol	eSNP	chromosome	coordinates	cis or trans	eQTL p-value	GWAS p-value	LBF
MYCN	rs1430059	2	16581896	cis	1.51E-04	1.40E-03	2.03E+00
MYCN	rs9823217	3	19340696	trans	3.63E-07	3.26E-01	-2.78E-03
MYCN	rs1860230	5	142077863	trans	6.49E-07	9.54E-01	-4.00E-03
MYCN	rs10795688	10	5983762	trans	4.74E-07	6.07E-01	-1.03E-02
MYCN	rs112917620	11	72384926	trans	1.31E-08	8.94E-01	-4.49E-02
MYCN	rs17426239	12	116438287	trans	5.50E-07	7.83E-01	-8.69E-03
MYCN	rs116184459	14	21059722	trans	2.74E-07	6.45E-02	1.42E-02
MYCN	rs2858006	16	297671	trans	8.13E-07	9.31E-01	-5.40E-03

Gene Symbol	Total LBF Score	P-value
TRIM4	1.96096	0.000943096

Gene Symbol	eSNP	chromosome	coordinates	cis or trans	eQTL p-value	GWAS p-value	LBF
TRIM4	rs72878574	2	180994194	trans	9.35E-07	1.60E-01	-6.53E-04
TRIM4	rs2572011	7	99487071	cis	4.23E-10	2.54E-04	1.96E+00
TRIM4	rs35266651	10	82013022	trans	9.79E-07	7.06E-01	-5.64E-04

Gene Symbol	Total LBF Score	P-value
ZNF57	1.94342	0.000963839

Gene Symbol	eSNP	chromosome	coordinates	cis or trans	eQTL p-value	GWAS p-value	LBF
ZNF57	rs2960820	7	25601480	trans	9.87E-07	9.80E-01	-3.19E-03

ZNF57	rs2060277	19	2925159	cis	1.67E-04	1.51E-03	1.95E+00

**Gene Symbol**	**Total LBF Score**	**P-value**
NARS2	1.93558	0.000973519

**Gene Symbol**	**eSNP**	**chromosome**	**coordinates**	**cis or trans**	**eQTL p-value**	**GWAS p-value**	**LBF**
NARS2	rs1813008	11	78196411	cis	2.51E-05	1.54E-04	1.94E+00
